# Application of a new approach methodology (NAM)-based strategy for genotoxicity assessment of data-poor compounds

**DOI:** 10.3389/ftox.2023.1098432

**Published:** 2023-01-23

**Authors:** Anne-Marie V. Fortin, Alexandra S. Long, Andrew Williams, Matthew J. Meier, Julie Cox, Claire Pinsonnault, Carole L. Yauk, Paul A. White

**Affiliations:** ^1^ Department of Biology, University of Ottawa, Ottawa, ON, Canada; ^2^ Environmental Health Science and Research Bureau, Health Canada, Ottawa, ON, Canada; ^3^ Existing Substances Risk Assessment Bureau, Health Canada, Ottawa, ON, Canada; ^4^ Bureau of Gastroenterology, Infection and Viral Diseases, Health Canada, Ottawa, ON, Canada; ^5^ New Substances Assessment and Control Bureau, Health Canada, Ottawa, ON, Canada

**Keywords:** genetic toxicology, new approach methodologies (NAM), TGx-DDI transcriptomic biomarker, human health risk assessment, data-poor compounds, toxicogenomics (TGx), multiflow, microflow

## Abstract

The conventional battery for genotoxicity testing is not well suited to assessing the large number of chemicals needing evaluation. Traditional *in vitro* tests lack throughput, provide little mechanistic information, and have poor specificity in predicting *in vivo* genotoxicity. New Approach Methodologies (NAMs) aim to accelerate the pace of hazard assessment and reduce reliance on *in vivo* tests that are time-consuming and resource-intensive. As such, high-throughput transcriptomic and flow cytometry-based assays have been developed for modernized *in vitro* genotoxicity assessment. This includes: the TGx-DDI transcriptomic biomarker (i.e., 64-gene expression signature to identify DNA damage-inducing (DDI) substances), the MicroFlow^®^ assay (i.e., a flow cytometry-based micronucleus (MN) test), and the MultiFlow^®^ assay (i.e., a multiplexed flow cytometry-based reporter assay that yields mode of action (MoA) information). The objective of this study was to investigate the utility of the TGx-DDI transcriptomic biomarker, multiplexed with the MicroFlow^®^ and MultiFlow^®^ assays, as an integrated NAM-based testing strategy for screening data-poor compounds prioritized by Health Canada’s New Substances Assessment and Control Bureau. Human lymphoblastoid TK6 cells were exposed to 3 control and 10 data-poor substances, using a 6-point concentration range. Gene expression profiling was conducted using the targeted TempO-Seq™ assay, and the TGx-DDI classifier was applied to the dataset. Classifications were compared with those based on the MicroFlow^®^ and MultiFlow^®^ assays. Benchmark Concentration (BMC) modeling was used for potency ranking. The results of the integrated hazard calls indicate that five of the data-poor compounds were genotoxic *in vitro*, causing DNA damage *via* a clastogenic MoA, and one *via* a pan-genotoxic MoA. Two compounds were likely irrelevant positives in the MN test; two are considered possibly genotoxic causing DNA damage *via* an ambiguous MoA. BMC modeling revealed nearly identical potency rankings for each assay. This ranking was maintained when all endpoint BMCs were converted into a single score using the Toxicological Prioritization (ToxPi) approach. Overall, this study contributes to the establishment of a modernized approach for effective genotoxicity assessment and chemical prioritization for further regulatory scrutiny. We conclude that the integration of TGx-DDI, MicroFlow^®^, and MultiFlow^®^ endpoints is an effective NAM-based strategy for genotoxicity assessment of data-poor compounds.

## Introduction

There is an urgent need to improve the efficiency and predictivity of the existing toxicology testing paradigms to address the immense backlog of chemicals requiring evaluation by regulatory bodies worldwide (USNRC, 2007; Schoeters, 2010; Barton-Maclaren et al., 2017; Council of Canadian Academies, 2017). One of the main challenges is the large number of chemicals with no or very limited experimental toxicology data, known as “data-poor compounds” (Barton-Maclaren et al., 2017). To address the paucity of experimental data, integrated data streams from higher-throughput methodologies are being applied for chemical prioritization and risk assessment. This includes New Approach Methodologies (NAMs), defined broadly as *in silico, in chemico* and *in vitro* assays, that avoid the use of animals to identify chemical hazards (ECHA, 2016; Kavlock et al., 2018). NAMs seek to modernize traditional toxicology testing strategies by addressing the current limitations with conventional assays to accelerate the pace of hazard assessment and reduce reliance on animal tests that are time-consuming and resource-intensive (Pfuhler et al., 2014; Kavlock et al., 2018).

Genotoxicity testing is a critical component of all chemical hazard and risk assessments. Chemicals that induce genetic damage may cause long-term adverse health outcomes including cancer, heritable genetic disorders, and other degenerative conditions (Phillips and Arlt, 2009; Heflich et al., 2020). The conventional genotoxicity test battery generally includes a bacterial reverse mutation test (i.e., Ames test) and an *in vitro* mammalian cell chromosomal damage test and/or mutation assay (ICH, 1998; USFDA, 2007; OECD, 2017). Depending on the results obtained and/or decision-making context, one or more *in vivo* tests may also be required (ICH, 1998; Thybaud et al., 2007; OECD, 2017). However, this test battery is not well suited to assessing the large number of data-poor chemicals needing evaluation. Most of the *in vitro* tests used are generally lower-throughput, provide little mechanistic information, and have a limited ability to predict effects *in vivo*. Chromosome damage assays, in particular, often yield positive calls for chemicals that do not pose an appreciable mutagenic risk to humans; these hazard calls can lead to unnecessary, costly, and time-consuming *in vivo* follow-up (Kirkland et al., 2006; Kirkland et al., 2007; Thybaud et al., 2007; Nesslany, 2017). Thus, in recent years there have been efforts to develop modernized *in vitro* genotoxicity testing tools that address these limitations. To meet this demand, toxicogenomic (TGx) and flow cytometric-based approaches have been developed for high-throughput and high-content assessment of DNA damage in human cells.

Transcriptional profiling can identify early molecular markers of toxicological mode of action (MoA) and/or effects (Thomas et al., 2013; Farmahin et al., 2017; Yauk et al., 2019). Since analysis of the large data sets produced by transcriptomics is complex, transcriptomic biomarkers have been developed to more efficiently and objectively predict toxicity from these data (Krewski et al., 2020). For example, Li et al. (2015) developed the TGx-DDI transcriptomic biomarker. The TGx-DDI biomarker is comprised of 64 genes; it is used to classify chemicals as DNA damage-inducing (DDI) or non-DDI by analyzing changes in gene expression following *in vitro* exposure of cultured mammalian cells. The 64 genes were identified from gene expression profiles of human TK6 cells exposed to a reference set of 28 chemical agents spanning a wide range of known DDI and non-DDI mechanisms including aneugenicity (i.e., change in chromosome number) (Li et al., 2015). The biomarker has been extensively validated and confirmed to be amenable to use with numerous gene expression technologies (Li et al., 2017; Cho et al., 2019; Buick et al., 2021). A variety of proof-of-concept studies have shown its potential utility for hazard identification, chemical prioritization, and potency comparisons (Buick et al., 2015; Moffat et al., 2015; Li et al., 2017; Cho et al., 2019; Li et al., 2019; Buick et al., 2020; Buick et al., 2021).

A series of miniaturized flow cytometry-based assays have been developed to accelerate *in vitro* genotoxicity testing [Bryce et al. (2008), Bryce et al. (2010), Bryce et al. (2013), Bryce et al. (2014), Bryce et al. (2017)]. The *in vitro* MicroFlow^®^ assay applies an automated approach to score micronucleus (MN) frequency to identify chromosomal damage induced by chemicals (Bryce et al., 2008; Bryce et al., 2010; Bryce et al., 2013). This approach has been thoroughly validated and is now part of existing test guidelines (TG 487) as an option for MN scoring (OECD, 2017). The MultiFlow^®^ DNA Damage assay uses several multiplexed biomarker responses to further classify genotoxic activity based on the MoA. This includes the measurement of: phosphorylation of histone H2AX (ɣH2AX), which is indicative of DNA double strand breaks; phosphorylation of histone H3 (p-H3) to identify mitotic cells; nuclear p53 localization to identify DNA damage responses; and the frequency of 8n DNA content to detect polyploidization (Bryce et al., 2014; Bryce et al., 2016; Bryce et al., 2017). Responses in specific MultiFlow^®^ biomarkers can classify genotoxic agents into two main MoAs: 1) clastogenicity, which is characterized by chromosomal insertions, deletions, or rearrangements that form *via* breakage (i.e., DNA double stand breaks); or 2) aneugenicity.

Published case studies have explored the integration of the aforementioned assays to assess the genotoxicity of prototypical compounds with promising results (Buick et al., 2020; Smart et al., 2020; Avlasevich et al., 2021). Buick et al. (2020) applied the TGx-DDI biomarker in parallel with the MicroFlow^®^ assay in HepaRG™ cells (Buick et al., 2020). Avlasevich et al. (2021) evaluated the performance of the *in vitro* MicroFlow^®^ and MultiFlow^®^ genotoxicity endpoints (Avlasevich et al., 2021). These case studies demonstrate that integrating several *in vitro* genotoxicity assays provides a more robust and accurate hazard assessment, with a limited number of irrelevant positives. As such, all three assays have been incorporated into the GeneTox21 research program at Health Canada to establish an effective *in vitro* platform for genotoxicity assessment (Felter et al., 2021).

Herein we explore the utility of the TGx-DDI transcriptomic biomarker, multiplexed with the *in vitro* MicroFlow^®^ assay and the MultiFlow^®^ DNA damage assay, as a NAM-based integrated test strategy for assessing the genotoxicity of data-poor compounds prioritized by Health Canada’s New Substances Assessment and Control Bureau (NSACB). The data-poor compounds were flagged by regulatory partners as having carcinogenicity/genotoxicity structural alerts using the Organisation for Economic Co-operation and Development (OECD) Quantitative Structure-Activity Relationships (QSAR) Toolbox (Dimitrov et al., 2016). Human lymphoblastoid TK6 cells were exposed to ten NSACB data-poor substances in conjunction with two positive and one negative control chemicals. Cells were exposed with or without exogenous metabolic activation (i.e., rat liver S9) to a range of concentrations. Gene expression profiling was conducted using the targeted TempO-Seq™ assay (Yeakley et al., 2017; Mav et al., 2018) and the TGx-DDI classifier was applied to the dataset. Classifications were compared with those based on the MicroFlow^®^ and MultiFlow^®^ assays. Benchmark Concentration (BMC) modeling of the genotoxicity endpoints was used to rank the test chemicals by their potencies. The Toxicological Prioritization (ToxPi) software (Reif et al., 2013; Marvel et al., 2018) was used to integrate the multiplexed endpoint BMCs and create a visual toxicological profile. Overall, we demonstrate how this novel NAM-based *in vitro* testing strategy provides a robust and efficient approach to identify genotoxic data-poor substances and assess potency for further prioritization.

## Materials and methods

### Chemicals investigated

Test chemical information (including the solvent control) and their respective abbreviations, CAS RN, and source are presented in Table 1.

**TABLE 1:** Information on test chemicals used in this study.

Chemical	Abbreviation	CAS no.	Source
2-amino-5-chlorobenzophenone	NSACB 1	719-59-5	Sigma-Aldrich (Oakville, ON)
2-amino-4-methylphenol	NSACB 2	95-84-1	Sigma-Aldrich (Oakville, ON)
3,5-dimethylpyrazole-1-methanol	NSACB 3	85264-33-1	Sigma-Aldrich (Oakville, ON)
3-diethylaminophenol	NSACB 4	91-68-9	Sigma-Aldrich (Oakville, ON)
2,6-diaminopyridine	NSACB 5	141-86-6	Sigma-Aldrich (Oakville, ON)
2-methoxy-4-nitrophenol	NSACB 6	3251-56-7	Sigma-Aldrich (Oakville, ON)
2-[(3-amino-4-methoxyphenyl)amino]ethanol	NSACB 7	83763-47-7	Best of Chemicals (BOC) Sciences (Shirley, NY)
2-[(4-methyl-2-nitrophenyl)amino]ethanol	NSACB 8	100418-33-5	Toronto Research Chemicals Inc (North York, ON)
4-[(3-hydroxypropyl)amino]-3-nitrophenol	NSACB 9	92952-81-3	Toronto Research Chemicals Inc (North York, ON)
1-(1-methyl-2-propoxyethoxy)-2-propanol	NSACB 10	29911-27-1	Sigma-Aldrich (Oakville, ON)
7,12-dimethylbenz[a]anthracene	DMBA	57-97-6	Sigma-Aldrich (Oakville, ON)
Etoposide	EPEG	33419-42-0	Sigma-Aldrich (Oakville, ON)
D-mannitol	DMANN	69-65-8	Sigma-Aldrich (Oakville, ON)
Dimethyl sulfoxide	DMSO	67-85-5	Sigma-Aldrich (Oakville, ON)

### Cell culture

All experiments were performed with human lymphoblastoid TK6 (IVTG strain) cells (ECACC #13051501) purchased from Sigma-Aldrich Canada (Oakville, ON). Cells were grown in RPMI1640 cell culture medium (ThermoFisher Scientific, Ottawa, ON) supplemented with 10% horse serum (ThermoFisher Scientific, Ottawa, ON), 2 mM L-glutamine (ThermoFisher Scientific, Ottawa, ON), 1.8 mM sodium pyruvate (ThermoFisher Scientific, Ottawa, ON), and 100 U/mL of penicillin and streptomycin (ThermoFisher Scientific, Ottawa, ON). Cells were incubated at 37°C with 5% CO_2_ and maintained below 1 x 10^6^/mL and >90% viability.

### Viability assessment

Cells were adjusted to a density of 1.5 x 10^5^/mL, aliquoted in 96-well plates, and exposed to 1 µL of test chemical (1% v/v, final volume 100 µL) solubilized in DMSO at a range of concentrations. For test chemicals requiring metabolic activation the medium was supplemented with 10 µL of 5% S9 Mix (0.5% v/v) containing Aroclor-1254 Induced Mutazyme^®^ (Moltox, Boone, NC). Treated cells were then incubated at 37°C with 5% CO_2_ for 4H. Cells were collected *via* centrifugation at 300 x g for 5 min, washed with Dulbecco’s phosphate buffered saline (DPBS) (ThermoFisher Scientific, Ottawa, ON), resuspended in fresh media, and incubated at 37°C with 5% CO_2_ for 20 h. The viable cell count was quantified *via* propidium iodide fluorescence analysis with the Miltenyi Biotec MACSQuant ^®^ Analyzer 10 flow cytometer with integrated 96-well MiniSampler device. Instrument settings: autolabel PI. The fluidics parameters were as follows: sample volume of 100 μL at a medium mix rate, followed by a sample uptake volume of 25 µL analyzed at a medium flow rate. The screen mode was used to rinse the probe between samples. Top concentrations were selected (Table 2) up to 10 mM or lower due to solubility (OECD, 2016). If cytotoxic, top concentrations were selected for each assay that induce: approximately 40% viability (i.e., 60% cytotoxicity) for the TGx-DDI assay and *in vitro* MicroFlow^®^ assay (Bryce et al., 2008; OECD, 2016), and 20% viability (i.e., 80% cytotoxicity) for the MultiFlow^®^ DNA Damage assay (Bryce et al., 2016).

**TABLE 2:** Experimental information for the data-poor chemicals and controls used in this study.

Chemical	S9 condition	TGx-DDI^a^	MultiFlow/MicroFlow^a^
		Top concentration–Lowest concentration	Top concentration–Lowest concentration
		6 concentrations (70.71% spacing)	10 concentrations (70.71% spacing)
NSACB 1	-S9	135–24.0 µM	382–16.9 µM
	+S9	95.5–16.9 µM	270–11.9 µM
NSACB 2	-S9	-	884–39.0 µM
	+S9	156–28.0 µM	884–39.0 µM
NSACB 3	-S9	-	884–46.0 µM
	+S9	65–12 µM	185–8.00 µM
NSABC 4	-S9	-	1250–55.00 µM
	+S9	55–10 µM	1768–78.00 µM
NSACB 5	-S9	-	10000–442.00 µM
	+S9	5000–884.0 µM	10000–442.00 µM
NSACB 6	-S9	-	10000–442.00 µM
	+S9	5000–884.0 µM	10000–442.00 µM
NSACB 7	-S9	35–6.0 µM	142–6.30 µM
	+S9	100.1–17.70 µM	400–17.7 µM
NSACB 8	-S9	1250–221.0 µM	3536–156.3 µM
	+S9	1250–221.0 µM	3536–156.3 µM
NSACB 9	-S9	2356–417.0 µM	6665–295.0 µM
	+S9	2600–460.0 µM	6665–295.0 µM
NSACB 10	-S9	10000–1768.0 µM	10000–442.00 µM
	+S9	10000–1768.0 µM	10000–442.00 µM
EPEG	-S9	0.16–0.028 µM	0.63–0.028 µM
	+S9	-	0.63–0.028 µM
DMBA	-S9	-	750–33.0 µM
	+S9	4.1–0.73 µM	23.4–1.04 µM
DMANN	-S9	10000–1768.0 µM	10000–442.00 µM
	+S9	10000–1768.0 µM	10000–442.00 µM
AMP	-S9	4957–876.0 µM	4957–219.0 µM
	+S9	4957–876.0 µM	4957–219.0 µM

^a^ Concentrations with <20% viability for MultiFlow^®^ and <40% viability for TGx-DDI, and MicroFlow^®^ assays were excluded from analysis.

### Exposure for TGx-DDI assay

Cells were adjusted to a density of 1.5 x 10^5^/mL, aliquoted in 96-well plates, and exposed to 1 µL of test chemical (1% v/v, final volume 100 µL/well) solubilized in DMSO in a six-point concentration range on a half-log distribution scale in duplicate (Table 2). Six DMSO solvent controls were included per plate. For test chemicals requiring metabolic activation, the medium was supplemented with 10 µL of 5% S9 Mix (0.5% v/v) containing Aroclor-1254 Induced Mutazyme^®^ (Moltox, Boone, NC). Treated cells were incubated at 37°C with 5% CO_2_ for 4H. Cells were collected *via* centrifugation at 300 x g for 5 min, washed with DPBS, and resuspended in 1X TempO-Seq™ Enhanced Lysis buffer (BioSpyder Technologies, Carlsbad, CA, United States) in DPBS (40 µL/well) (ThermoFisher Scientific, Ottawa, ON). If treated with 0.5% rat liver S9, cells were collected *via* centrifugation at 300 x g for 5 min, washed with DPBS (ThermoFisher Scientific, Ottawa, ON), resuspended in fresh media, and incubated at 37°C with 5% CO_2_ for a 3 h recovery period. Based on preliminary data select compounds were only tested in one metabolic condition. Following incubation, cells were collected, washed, and resuspended in 1X TempO-Seq™ Enhanced Lysis buffer (BioSpyder Technologies, Carlsbad, CA, United States) in DPBS (ThermoFisher Scientific, Ottawa, ON) (40 µL/well) and frozen at −80°C.

### TempO-Seq™, library purification and sequencing

The TempO-Seq™ Human Surrogate + Tox Panel (S1500+) v2.0 (BioSpyder Technologies, Carlsbad, CA, United States) assay was completed following the manufacturer’s instructions in a 96-well plate format. Each 96-well plate included three assay controls in duplicate: a negative no-cell lysate (1X TempO-Seq™ Enhanced Lysis buffer only), Human Reference Total RNA (Takara Bio, CA, United States), and Human Brain Reference Total RNA (Takara Bio, CA, United States). Briefly, for each treatment, 2 µL of cell lysate in 1X TempO-Seq™ Enhanced Lysis buffer was hybridized to the Human S1500 + Surrogate detector oligo (DO) probe mix (V2.0), incubated at 70°C for 10 min, then ramped down to 45°C over 50 min (0.5°C/min). A nuclease digestion followed to remove excess or incorrectly bound DOs at 37°C for 1 h. The bound DO pairs were then ligated together at 37°C for 1 h and 15 min at 80°C to generate templates for amplification. A 10 µL aliquot of amplification template was transferred to its respective well in the TempO-Seq™ PCR Pre-Mix and Primers plate and amplified on the CFX96 thermocycler (Bio-Rad, Mississauga, ON, Canada) with the following program: 37°C for 10 min, 95°C for 1 min; 25 cycles of 95°C for 10 s, 65°C for 30 s, 68°C for 30 s; 68°C for 2 min. For library building and purification, three 96-well plates (288 sample libraries) were pooled together (5 µL per sample) and purified using the Macherey-Nagel NucleoSpin^®^ Gel and PCR Cleanup Kit (Clontech Laboratories Inc., Bethlehem, PA, United States) with the adjustments specified by the TempO-Seq™ Assay User Guide. The pooled purified TempO-Seq™ libraries were then diluted, quantified, and assessed for quality using the Agilent High Sensitivity D1000 TapeStation (Agilent Technologies, Santa Clara, CA, United States) and the qPCR KAPA Library Quantification Kit (Universal qPCR Master Mix) for Illumina NextSeq 500. Samples were sequenced on a total of two NextSeq^®^ 500/550 High Output (75-cycle) flow cells using an Illumina NextSeq^®^ 500 Sequencing Platform (Illumina, San Diego, CA, United States). A separate pool was completed for a fourth plate and an additional 96 sample libraries were added to the sequencing data for subsequent analysis.

### Sequencing data preprocessing and alignment

Sequencing data are accessible in the National Centre for Biotechnology Information (NCBI) Gene Expression Omnibus (GEO) database under accession number GSE213454. Briefly, bcl2fastq (v2.20.0.422) was used to demultiplex the raw sequencing data and assign each data set to its respective sample files. The data were filtered using fastp (v0.20.0) to eliminate reads shorter than 50 bp and remove low-quality sequences. The BioSpyder TempO-SeqR v3.0 analysis script, implemented as part of our transcriptomics data processing pipeline (https://github.com/R-ODAF/R-ODAF_Health_Canada), was used to align the FASTQ files for each sample to the reference sequences for the TempO-Seq™ Human Surrogate + Tox Panel (S1500+) v2.0 probes producing a table of counts per probe per sample. A study-wide quality control analysis workflow adapted from Harrill et al. (2021) was applied to the count matrix; 7/276 samples (i.e., S1_pool2, S2_pool2, S31, S79, S95_pool2, S109, S119) were removed due to low quality.

### Statistical analyses for TGx-DDI classification

The count matrix underwent log2(CPM +1) normalization to account for read-depth variability between samples. TGx-DDI genes with multiple probes were averaged. For each compound, sample replicates were averaged and the log2 fold changes were estimated for each concentration. Samples that were cytotoxic (viability <40%), or did not achieve a minimum read depth, were removed from the analysis.

Detailed information regarding the TGx-DDI classification statistical analyses has been described previously (Yauk et al., 2016; Buick et al., 2017; Buick et al., 2021). Briefly, to classify chemicals as DDI or non-DDI, a three-pronged statistical approach was applied. Each analysis compares the sample biomarker responses to those of training set compounds with known DDI and non-DDI mechanisms. The three analyses are listed below.

1) The Nearest Shrunken Centroids Probability Analysis (NSC-PA) method was conducted (Tibshirani et al., 2002) using the pamr() function in R. This summarized the training dataset by calculating a standardized centroid (SC) for the DDI and non-DDI chemicals in the training set. The SC represents the average expression for each gene in a class relative to its within-class standard deviation. To create the NSC, the SCs were shrunken in the direction of the overall centroid. To classify each concentration for each chemical, the expression profiles were compared to the training set NSCs (Li et al., 2017) and assigned to a class (DDI or non-DDI) based on the probability that class membership was >0.90 (else it was not classifiable). This analysis was visualized as a heatmap.
2) Principal Component Analysis (PCA) was conducted using the prcomp() function in R (Venables and Ripley, 2002). The PCA estimated the principal components (PC) of the training set data and the PCA loadings were applied to the experimental samples. The sample and training set data were visualized in a scatterplot. Chemical concentrations with a negative PC1 were classified as DDI and with a positive PC1 were classified as non-DDI.
3) 2-Dimentional hierarchical clustering (2-DC) based on average linkage with Euclidean distances (Becker et al., 1990) was generated for the training set and experimental data using the hclust() function in R. Clustering on the main branch with non-DDI agents or on the main branch with DDI agents led to non-DDI or DDI calls for those chemical concentrations, respectively. If the sample was on a branch outside the main DDI and non-DDI clusters, that concentration could not be classified.

If a chemical had a positive call in one of the three statistical analyses (NSC-PA, PCA, 2-DC) at any concentration, the overall call assigned was DDI. Conversely, if none of the three analyses produced a positive call, the overall call assigned was non-DDI.

### Exposure for the MultiFlow and MicroFlow assays

Cells were adjusted to a density of 2.0 x 10^5^/mL and cells were aliquoted in 96-well plates. Cells were exposed to 2 µL of solubilized test chemical (1% v/v, final volume 200 µL/well) in a ten-point concentration range on a half-log distribution scale in (Table 2). All chemicals were also tested in the presence of S9 applying the conditions described previously. Treated cells were incubated at 37°C with 5% CO_2_ for 4H. At the 4H timepoint, aliquots of cells were collected for the MultiFlow^®^ DNA Damage assay (Litron Laboratories, Rochester, NY, United States). The remaining cells were collected *via* centrifugation at 300 x g for 5 min, washed with DPBS (ThermoFisher Scientific, Ottawa, ON), resuspended in fresh media, and incubated at 37°C with 5% CO_2_. These cells were incubated for an additional 20H for the second time point of the MultiFlow^®^ DNA Damage assay and for the *in vitro* MicroFlow^®^ assay (Litron Laboratories, Rochester, NY, United States).

### *In vitro* MicroFlow^®^ assay processing and analysis

At 24-h post-exposure cells were collected *via* centrifugation at 300 x g for 6 min. The supernatant was carefully removed and freshly prepared Complete Nucleic Acid Dye A (50 µL/well) was added. The plate was then placed under a visible light source on ice for 30 min, 1X Buffer Solution (150 µL/well) was added, cells were collected *via* centrifugation at 300 x g for 6 min, and the supernatant was carefully removed. Cells were resuspended in Complete Lysis Solution 1 (100 µL/well), mixed thoroughly, and incubated at room temperature for 1 h. Completed Lysis Solution 2 was added (100 µL/well) and the plate was rocked gently to mix. The plate was then analyzed *via* flow cytometry with the Miltenyi Biotec MACSQuant^®^ Analyzer 10 flow cytometer with an integrated 96-well MiniSampler. The mixing and fluidics parameters were as follows: sample mixing of 50 μL at a medium mix rate, followed by a sample uptake volume of 50 µL that was analyzed at a medium flow rate. The fast mode was used to rinse the probe between samples. Instrument settings followed instructions stated in the MicroFlow^®^ MicroNucleus Analysis kit (*In Vitro*, 96 well) (Litron Laboratories, Rochester, NY) to detect fluorochromes SYTOX Green^®^ in the FITC channel and ethidium monoazide (EMA), in the PerCP-Cy5.5 channel. An analysis stop gate of 5,000 EMA-negative nuclei per well was applied.

The *in vitro* MicroFlow^®^ results were analyzed using the analysis template provided by Litron Laboratories (Rochester, NY). The % MN was calculated using the count of MN events relative to the count of nucleated events. Results for graphical and statistical representations were expressed as a relative fold-change from the average plate-specific DMSO solvent control normalized to 1.

$$\%\;MN=\frac{MN\;Events}{Nucleated\;Events}\;x\;100$$

Cytotoxicity was determined using the Relative Survival (RS) equation. Treatments with RS values less than 40% and %EMA-positive nuclei values greater than 4-fold over solvent controls were removed from analysis.

$$RS=\frac{Nuclei\;in\;treated\;culture/mL}{Nuclei\;in\;mean\;solvent\;control/mL}\;x\;100$$

$$Fold\;EMA=\frac{\%\;Apoptotic/Necrotic}{Mean\;\%\;Apoptotic/Necrotic\;of\;solvent\;controls}\;x\;100$$

A positive call was indicated by a 2.50-fold increase in %MN and a statistically significant (*p* < 0.05) %MN increase relative to solvent controls in at least one non-cytotoxic concentration (Avlasevich et al., 2021). Statistical significance was determined using a Poisson regression with a Holm-Sidak multiple correction procedure.

### MultiFlow DNA damage assay processing and analysis

At 4H and 24H sampling times, fresh complete labeling solution was prepared; solution containing nuclei release solution, DNA stain, RNase solution, ɣH2AX Alex Fluor^®^ 647, phosphor-histone antibody PE, and p53 antibody conjugated to fluorescein isothiocyanate (FITC) (Litron Laboratories, Rochester, NY) was added (50 µL/well) to a fresh 96-well plate. Treated cells were gently resuspended and 25 µL/well was mixed thoroughly with the labeling solution. The plate was incubated for 30 min at room temperature shielded from light. The cells were then analyzed *via* flow cytometry with the Miltenyi Biotec MACSQuant^®^ Analyzer 10 flow cytometer with an integrated 96-well MiniSampler. Instrument settings followed the MultiFlow^®^ Smart Start Guide (Litron Laboratories, Rochester, NY) to detect fluorescence emissions from fluorochromes FITC (in the B1 channel), PE (in the B2 channel), propidium iodide (in the B3 channel) and Alexa Fluor^®^ 647 (in the R1 channel). The mixing and fluidics parameters were as follows: sample mixing of 40 μL at a medium mix rate, followed by a sample uptake volume of 20 μL, which was analyzed at a medium flow rate. The fast mode was used to rinse the probe between samples (Bernacki et al., 2016; Bryce et al., 2016).

The MultiFlow ^®^ DNA Damage assay results were analyzed using the analysis template provided by Litron Laboratories (Rochester, NY). The γH2AX and p53 endpoints were measured based on their median fluorescence intensity (i.e., mean channel fluorescence or MCF) relative to the DMSO solvent control. The p-H3 and polyploidy endpoints were measured based on their frequencies among all events (i.e., all 2n–4n and polyploidy (8n) DNA content). The equations for calculating γH2AX shift, nuclear p53 shift, % polyploidy, and % p-H3 positive events are shown below. Results for graphical and statistical representations were expressed as a relative fold-change from the average plate-specific DMSO solvent control normalized to 1 (Bernacki et al., 2016; Bryce et al., 2016).

$$\gamma H2AX\;shift=\frac{MCF\;of\;treated\;culture}{Mean\;MCF\;of\;solvent\;control}$$

$$Nuclear\;p53\;shift=\frac{MCF\;of\;treated\;culture}{Mean\;MCF\;of\;solvent\;control}$$

$$\%\;Polyploidy=\frac{\#\;events\;polyploidy\;\left(8n\right)\;DNA\;content}{\#\;events\;2n,\;4n\;and\;polyploidy\;DNA\;content\;}\;x\;100$$

$$\%\;pH3\;events=\frac{\#\;events\;pH3\;positive\;4n\;and\;greater\;DNA\;content}{\#\;events\;2n,\;4n\;and\;polyploidy\;DNA\;content\;}\;x\;100$$

Latex microspheres were used as counting beads to determine nuclear density. Cytotoxicity was determined using the Relative Nuclei Count (RNC) equation, with cytotoxicity = 100%—RNC at 24H (Bernacki et al., 2016; Bryce et al., 2016):

$$RNC=\frac{Density\;of\;nuclei\;in\;treated\;culture}{Density\;of\;nuclei\;in\;mean\;sovent\;control}\;x\;100$$

Litron developed Global Evaluation Factors (GEF) to identify a biologically significant increase in select biomarker levels. The determined GEFs, expressed as fold-change (FC) increase over solvent control, are as follows (Bryce et al., 2017).

• For clastogenic biomarkers: 4H γH2AX, 1.51 FC; 24H γH2AX, 2.11 FC; 4H p53, 1.40 FC; 24H p53, 1.45 FC.
• For aneugenic biomarkers: 4H p-H3, 1.71 FC; 24H p-H3, 1.52 FC; 24H polyploidy, 5.86 FC; 24H p53, 1.45 FC.

A clastogenic or aneugenic call required two successive concentrations that met or exceeded the GEFs in at least 2 of the 4 respective MoA biomarkers. Chemicals that met or exceeded two GEFs in a single non-cytotoxic concentration were designated as “weak” responses. In cases where both clastogen and aneugen biomarkers exceeded the GEF the MoA was considered pan-genotoxic. In cases where less than two biomarkers met the GEFs, the call was non-genotoxic. Exposure concentrations that resulted in greater than 80% cytotoxicity were excluded from MoA classification.

### Benchmark concentration modeling of TGx-DDI biomarker genes

Gene expression data were used for BMC modeling as described by Buick et al. (2021). Briefly, the read counts were log_2_ normalized. Groups where *N* = 1 were removed and filtered to exclude probes with fewer than 5 reads. The log_2_ transformed data were run with the BMDExpress V2.3 software following the guidelines outlined by the US National Toxicology Program (NTP) Approach to Genomic Dose-Response Modeling report (USNTP, 2018). The log2 data were prefiltered using a Williams Trend Test applying a permutation *p*-value cutoff <0.05 with 500 permutations and linear fold change of ≥1.5. The pre-filtered data were analyzed using EPA BMDS parametric models to derive BMCs. This included: the Exponential 2, 3, 4, and 5 models, Linear, 2° Polynomial, and the Restricted Power (≥1). The benchmark response factor (BMR) was set to 1 standard deviation. The “Best BMC” was selected based on the best-fit model [i.e., model with the lowest Akaike Information Criterion (AIC)]. The upper (BMCU) and lower (BMCL) 95% confidence limits of the BMCs were calculated. The calculated gene BMCs were filtered and removed if: the model fit was insufficient (*p*-value <0.1), BMC/BMCL ratio ≥20, BMCU/BMC ≥20, or BMCU/BMCL ≥40. Applying a bootstrapping method described in detail by Buick et al. (2021), the median BMC of the TGx-DDI biomarker genes was derived to represent an overall TGx-DDI BMC for each compound. The 95% confidence intervals were calculated from the bootstrapped distribution of the BMC median.

### Benchmark concentration modeling of MultiFlow^®^ and MicroFlow^®^ endpoints

BMC modeling was done on test chemicals with positive MultiFlow^®^ and/or MicroFlow^®^ calls. The modeling was performed with the PROAST R package (V70.3) developed by the RIVM (i.e., Dutch National Institute for Public Health and the Environment). To model the continuous concentration-response data, a single 5-parameter exponential model (y = a*[c^(1−exp(−x/b)^d)^]) was applied (White et al., 2020). For the MicroFlow^®^ assay, the % induction of MN relative to test chemical concentration was modeled; a BMR of 1.0 (i.e., 100% increase in response relative to control) was applied (Long et al., 2018; White et al., 2020; Avlasevich et al., 2021). For the MultiFlow assay, the fold change in response for each biomarker (e.g., 4H and 24H p53; 4H and 24H ɣH2AX) relative to test chemical concentration was modeled. A BMR of 0.5 (i.e., 50% increase) was applied (Avlasevich et al., 2021). The lowest BMC of the MultiFlow biomarkers was selected as the overall BMC.

### ToxPi visualization

The calculated endpoint BMCs were integrated and converted into a single visual profile for each compound using the Toxicological Prioritization Index (ToxPi) software (v2.3) (Reif et al., 2013; Marvel et al., 2018). The BMC, BMCL, and BMCU values for each compound were formatted in Excel using the templates provided by ToxPi and saved as a comma-separated value (csv) file. A value of 10,000 µM (i.e., 10 mM) was entered when an infinite upper confidence interval or a BMC could not be calculated for a particular endpoint; or if a calculated BMC value was greater than the actual top passing/valid concentration. Differential weighting was assigned to the slices. TGx-DDI and MN were assigned 1/3 of the profile each; the four p53 and ɣH2AX timepoints were assigned 1/12 of the profile each to make up 1/3. The BMCL and BMCU values were assigned to their respective slices and were −log_10_ transformed. The ToxPi analysis was run to derive ToxPi scores. First, the BMCL and BMCU values for each endpoint were summed and transformed into a single slice score for each compound. This was achieved by normalizing the summed BMC values to a [0, 1] interval by dividing by the slice maximum (USNRC, 2014). Thus, in this case, values closer to 1 (i.e., maximum unit score) denoted a higher potency. Alternatively, values closer to 0 (closer to the origin) denoted a lower potency; and values that did not extend from the origin were denoted as inactive. The overall ToxPi score was calculated by combining all weighted slice scores for each compound and the 95% confidence intervals were calculated. The ToxPi hierarchical clustering algorithm was applied to cluster like-chemical ToxPi profiles. ToxPi analysis figures (i.e., profiles, rank plot, and hierarchical clustering) were downloaded.

## Results

Human lymphoblastoid TK6 cells were exposed to ten data-poor chemicals across a range of concentrations alongside solvent controls, as well as three reference control chemicals. Genotoxicity was assessed using three *in vitro* assays: 1) the TGx-DDI transcriptomic biomarker, 2) the *in vitro* MicroFlow^®^ assay, and 3) the MultiFlow^®^ DNA Damage assay. Potency ranking for chemical prioritization was conducted on chemicals with positive hazard flags using BMC modeling and compared across the assays. A single genotoxicological profile integrating all endpoint BMCs was created using the ToxPi software.

Prior to assessing genotoxicity, concentration ranges were identified using viability studies ± S9 to select top concentrations for each assay. Cell viability was quantified at 24 h *via* propidium iodide fluorescence analysis (Supplementary Figure S1). Both positive controls, EPEG and DMBA, caused a decline in viability; whereas, the negative control, DMANN, showed no decrease in viability. NSACB chemicals #1–9 caused concentration-dependent decreases in viability both ± S9. NSACB #10 did not impact viability up to the highest concentration of 10 mM.

### TGx-DDI biomarker classification

**FIGURE 1:**
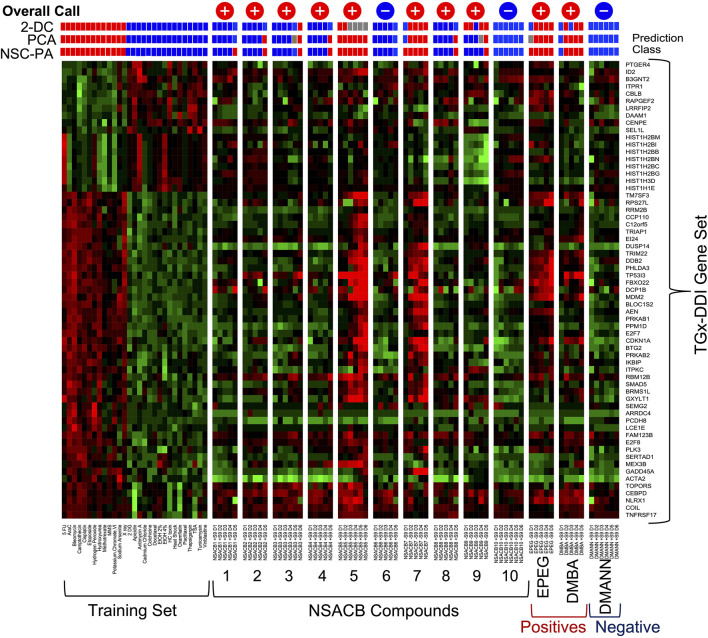
TGx-DDI classification of NSACB data-poor compounds. The heatmap on the left depicts the 28 reference chemicals used as a training set to generate the biomarker. The color scale indicates the average gene expression fold changes of two replicates relative to solvent control: up-regulated genes are shown in red, down-regulated genes are shown in green, genes with no change are shown in black. Three analyses: 1) 2-dimensional hierarchical clustering (2-DC), 2) principal component analysis (PCA), and 3) nearest shrunken centroid probability analysis (NSC-PA) were used to determine classification probabilities shown for all treatment conditions using red (DDI), blue (non-DDI), and grey (inconclusive) boxes. The overall calls are also shown at the top of each column: “+” signifies a positive DDI call, “−” signifies a non-DDI call. D1 represents the lowest concentration tested, D6 the highest. Cytotoxic concentrations (<40% relative survival) were removed from the analysis. The results are presented −S9 for compounds that had a positive DDI call in −S9 conditions (i.e., #7, 9, EPEG). All other results are presented +S9 (i.e., #1, 2, 3, 4, 5, 6, 8, 10, DMBA, DMANN). Additional results can be found in Supplementary Figure S2.

To classify test compounds as DDI or non-DDI using the TGx-DDI biomarker, transcriptional profiles were generated using TempO-Seq™ S1500 + sequencing. A trio of independent statistical analyses, 2-DC, PCA, and NSC-PA, were used to derive the overall TGx-DDI classifications (Figure 1; Supplementary Figures S2, S3). To ensure a conservative assessment of genotoxic potential, if one of the analyses classified a test compound as DDI, the overall call given was DDI. The biomarker correctly classified the three control chemicals: DMBA and EPEG were classified as DDI; the negative control DMANN was classified as non-DDI.

Eight out of ten NSACB compounds were identified as DDI, with varying potencies. NSACB compounds #5 and #7 showed the strongest responses. The trio of statistical analyses identified NSACB #5 as DDI across all concentrations (2-DC was inconclusive at the highest four concentrations); and NSACB #7 was DDI across all concentrations except the lowest. The strength of this response can be visualized in Figure 1, i.e., when comparing the similarity of the heatmaps of NSACB #5 and #7 to those DDI compounds in the training set. NSACB #9 had a moderate DDI response; the 2-DC statistical analysis identified #9 as DDI for the majority of concentrations, but the NSC-PA and PCA analyses only identified this compound as DDI at the highest non-cytotoxic concentration. NSACB compounds #1, 2, 3, 4, and 8 were also classified as DDI. However, these were considered weaker DDI responses as these compounds were only positive at the highest non-cytotoxic concentrations and the DDI calls were not consistent across the statistical analyses (with the exception of #3). NSACB compounds #6 and 10 were classified as non-DDI; all three analyses yielded non-DDI calls across all concentrations.

### *In vitro* MicroFlow^®^ assay classification

**FIGURE 2:**
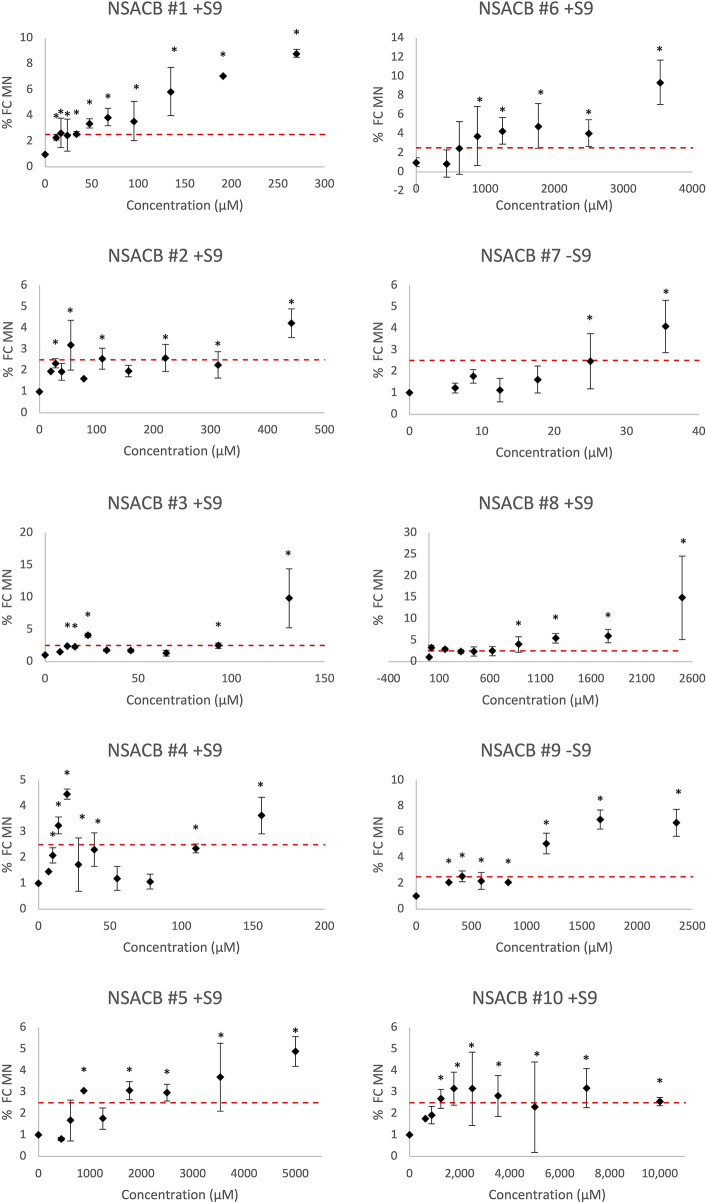
*In vitro* MicroFlow^®^ assay results for ten NSACB data-poor substances (#1–10). Fold-change in % micronucleus (% FC MN) compared to vehicle control is depicted by black diamonds. The dashed red line shows the threshold (i.e., 2.5-fold increase in %MN) required to yield a positive classification. Statistically significant (*p* < 0.05) increases in %MN in comparison with the concurrent vehicle control are designated by an asterisk (*). Cytotoxic concentrations (<40% viability) were removed from the analysis. Error bars denote standard deviation from mean. *N* = 2. For comparison, the S9 condition presented for each compound is as denoted in Figure 1. Additional results can be found in Supplementary Figure S4.

Chromosome damage was assessed using the *in vitro* MicroFlow^®^ assay for the test chemicals (Figure 2, Supplementary Figure S4). A positive result was designated by at least one concentration that induced >2.5 fold-increase in %MN and was statistically significant (*p* < 0.05) compared to vehicle controls. Both positive controls, EPEG and DMBA, exhibited significant fold-increases in %MN, whereas this was not observed with the negative control DMANN (Supplementary Figure S4).

All ten NSACB chemicals met the classification criteria for a positive call in at least one S9 condition (i.e., with/without S9) (Figure 2; Supplementary Figure S4). The majority were positive without S9, but #8 and #10 only tested positive with S9 conditions. NSACB #1, 5, 6, 7, 8, and 9 showed a robust fold-increase in %MN with a clear concentration-response relationship. NSACB #2 and #3 displayed variable responses with a significant increase observed only at the highest concentration. NSACB #4 and #10 exhibited a concentration-response pattern at the lowest concentrations before decreasing or plateauing at the higher concentrations.

### MultiFlow^®^ DNA damage assay classification

**FIGURE 3:**
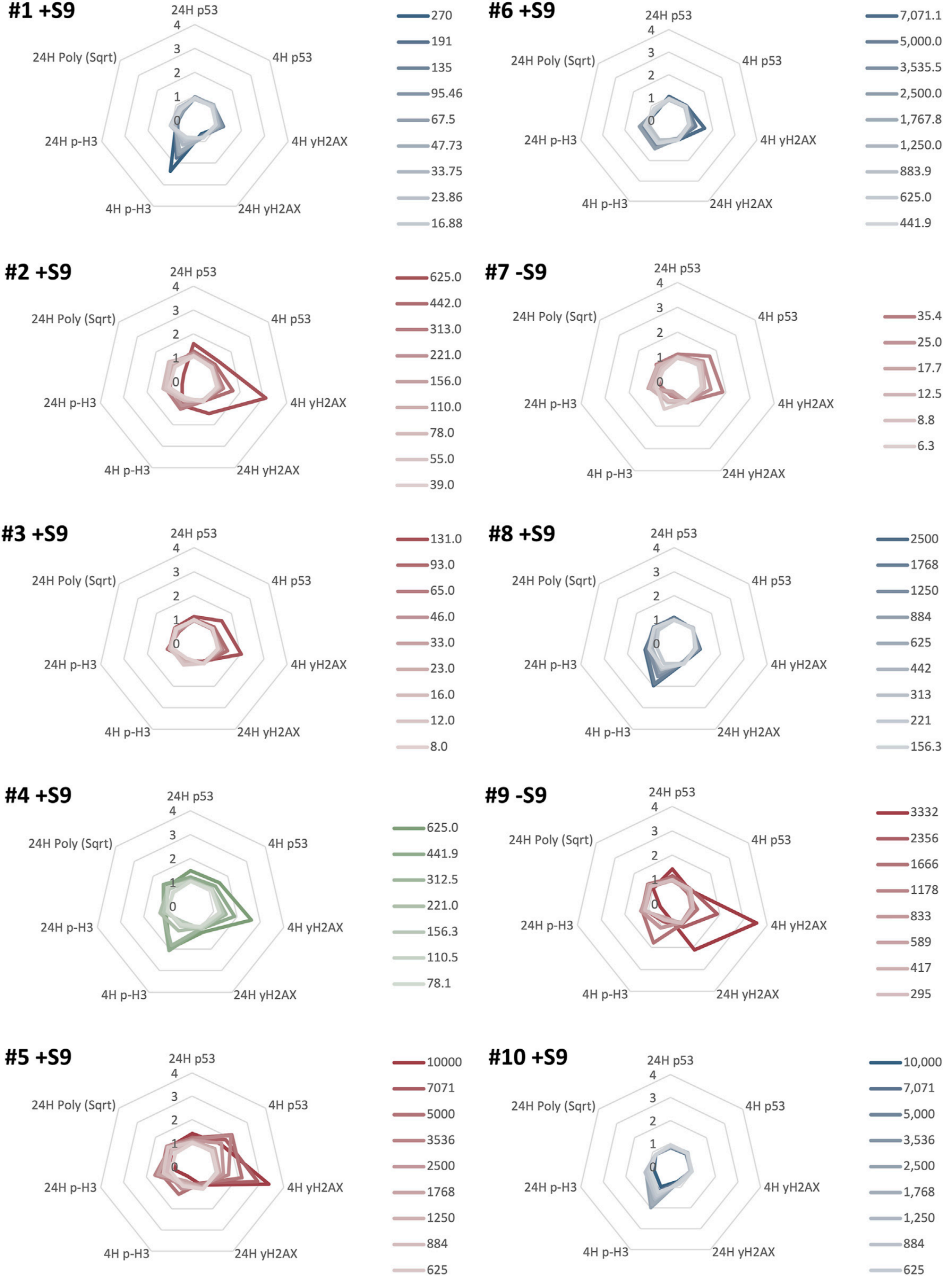
MultiFlow^®^ DNA Damage assay classification results for ten NSACB data-poor substances (#1–10). Each radar plot shows the seven biomarkers predicting the predominant mode of action (MoA) for each chemical. Clastogen MoA biomarkers are on the right of each radar plot: 4H p53, 4H ɣH2AX, 24H p53, and 24H ɣH2AX. Aneugen MoA biomarkers are on the left of each radar plot: 4H p-H3, 24H p-H3, 24H Polyploidy, and 24H p53. The biomarker data are expressed as a fold-increase over the mean vehicle control for each non-cytotoxic concentration (>20% viability) represented by lines with different colour intensities (as shown in the legend). The line colour in each plot represents the classification call: clastogens are red, non-genotoxicants are blue, and pan-genotoxicants are green. Chemicals meeting or exceeding the Global Evaluation Factors (GEFs) in at least one concentration in two MoA-specific biomarkers were classified as aneugenic or clastogenic, or classified as pan-genotoxic if both the aneugen and clastogen criteria were met. For comparison, the S9 condition presented for each compound is as denoted in Figures 1, 2. Additional results can be found in Supplementary Figure S5.

To provide insight into the MoA, the test compounds were assessed with the MultiFlow^®^ assay. The assay data were visualized using radar plots that depict the fold changes in the MultiFlow^®^ biomarkers following chemical exposure (Figure 3, Supplementary Figure S5). If a test chemical induced a significant increase in two out of four clastogen-specific biomarkers (i.e., 4H and 24H p53, and ɣH2AX), a clastogenic MoA was predicted. Similarly, significant responses in two out of four aneugen-specific biomarkers (4H p-H3, 24H p53, p-H3, and polyploidy) predicted an aneugenic MoA. If both of these criteria were met, a pan-genotoxic MoA was predicted. If none of these criteria were met the chemical was classified as non-genotoxic.

The control chemicals produced the expected results (Supplementary Figure S5). Positive controls, EPEG and DMBA, had concentration-dependent increases in the clastogen biomarkers (EPEG: 4H p53, 24H p53, and 4H ɣH2AX; DMBA: 4H & 24H ɣH2AX).

In total, six of the ten NSACB compounds were classified as genotoxic using the MultiFlow^®^ assay. NSACB compounds #2, 3, 5, 7, and 9 exhibited concentration-dependent increases in MultiFlow^®^ clastogen-specific biomarkers and were therefore classified as clastogens (Figure 3). NSACB #3, 5, and 7 induced responses in 4H p53 and 4H ɣH2AX; NSACB #2 induced responses in 4H ɣH2AX and 24H p53; and NSACB #9 induced responses in 4H and 24H ɣH2AX. NSACB #2, 5, and 9 had the most robust increases, whereas NSACB #3 and 7 had more moderate responses. NSACB #4 was the only test chemical classified as a pan-genotoxicant. This compound displayed significant increases in clastogen biomarkers 4H p53 and 4H ɣH2AX, as well as aneugen marker 4H p-H3 and pan-genotoxic biomarker 24H p53. The remaining compounds, NSACB #1, 6, 8, and 10 were classified as non-genotoxic. Three of these compounds (#1, 6, and 10) had a moderate to robust response increase in one of the aneugenic biomarkers (i.e., 4H p-H3) at more than one concentration. However, without an increase in any other biomarker, an aneugenic call could not be made.

### Summary of hazard calls

An overview of the TGx-DDI, MicroFlow^®^, and MultiFlow^®^ classification calls for the test chemicals is depicted in Table 3. The control chemicals performed as expected. EPEG and DMBA were positive across all three assays; DMANN tested negative in all three assays. For the data-poor compounds, eight out of ten (NSACB #1, 2, 3, 4, 5, 7, 8 and 9) were classified as DDI by the TGx-DDI transcriptomic biomarker in at least one S9 condition. In contrast, all ten NSACB compounds were positive for MN induction in at least one S9 condition. Using MultiFlow^®^, five were classified as clastogens and one was classified as a pan-genotoxicant in at least one S9 condition.

**TABLE 3:** Summary classifications for the ten data-poor compounds and control chemicals.

		#1	#2	#3	#4	#5	#6	#7	#8	#9	#10	EPEG	DMBA	DMANN
TGx-DDI	-S9	-						+	-	+	-	+		-
	+S9	+	+	+	+	+	-	+	+	+	-		+	-
MicroFlow^®^	-S9	+	+	+	+	+	+	+	-	+	-	+	+	-
	+S9	+	+	+	+	+	+	+	+	+	+	+	+	-*
MultiFlow^®^	-S9	-	-	+ C	+ C	+ C	-	+ C	-	+ C	-	+ C	-	-
	+S9	-	+ C	+ C	+ C/A	+ C	-	+ C	-	+ C	-	+ C	+ C	-

The classifications are as follows for each assay: red boxes with a “+” signify a positive call, blue boxes with a “-” signify a negative call, and dark grey boxes were not tested. For the MultiFlow^®^ DNA, Damage assay C, clastogen; A, aneugen; C/A, pan-genotoxicant. *Data leveraged from Litron Laboratories.

Overall, six compounds (i.e., NSACB #2, 3, 4, 5, 7, and 9) were positive in all three assays. NSACB #1 and #8 were positive in the TGx-DDI and MicroFlow^®^ assays, but were classified as non-genotoxicants by the MultiFlow^®^ assay. The remaining compounds, NSACB #6 and #10, only tested positive in the MicroFlow^®^ assay, and were classified as non-DDI and non-genotoxicants by the TGx-DDI and MultiFlow^®^ assays, respectively.

### Independent BMC analysis of TGx-DDI biomarker genes, MicroFlow^®^ and MultiFlow^®^ assay endpoints

**FIGURE 4:**
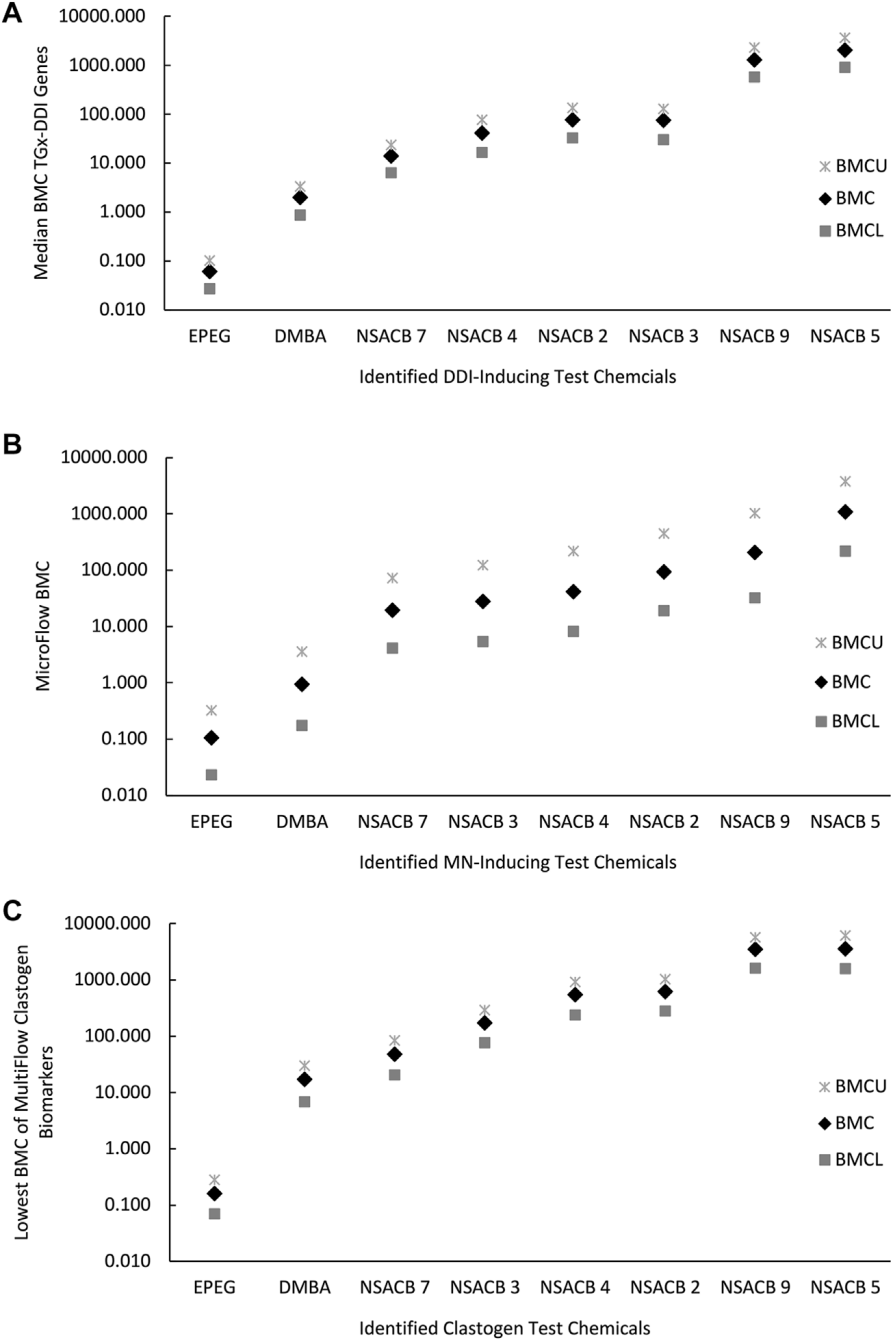
Comparison of potency rankings derived from each assay based on the respective Benchmark Concentrations (BMCs) for the NSACB compounds with concordant positive hazard flags. **(A)** The potency ranking from the TGx-DDI transcriptomic biomarker based on the bootstrapped median gene BMC, **(B)** the ranking from the *in vitro* MicroFlow^®^ assay, and **(C)** the potency ranking from MultiFlow^®^ assay based on the lowest clastogen biomarker BMC. The BMCU and BMCL represent the upper and lower 95% confidence limits of the BMC, respectively.

In addition to hazard calls, quantitative analyses of genotoxicity data can be applied for chemical potency ranking and subsequent prioritization. To assess the relative potency of the NSACB compounds with positive hazard flags, we conducted BMC modeling to derive BMC values for the TGx-DDI biomarker gene set, and the MicroFlow^®^ and MultiFlow^®^ assay endpoints (Supplementary Figure S6). To evaluate the consistency of the potency rankings derived from each assay, the NSACB compounds with concordant positive hazard calls (i.e., #2, 3, 4, 5, 7, and 9) were compared (Figures 4A–C). The most to least potent chemicals based on the TGx-DDI BMC ranking was (Figure 4A): EPEG (lowest median gene BMC) > DMBA > NSACB 7 > NSACB 4 > NSACB 2 > NSACB 3 > NSACB 9 > NSACB 5 (greatest median gene BMC). Two main groupings for the NSACB compounds can be observed. The first group, NSACB #7, 4, 3, and 2, have confidence intervals (CIs) that overlap and are thus are not significantly different from each other. The second group, NSACB #9 and #5, also have CIs that overlap, but are in a distinct, less potent, group from NSACB #7, 4, 3, and 2. MicroFlow^®^ BMC analysis yielded a highly similar potency ranking (Figure 4B): EPEG > DMBA > NSACB 7 > NSACB 3 > NSACB 4 > NSACB 2 > NSACB 9 > NSACB 5. However, due to the overlapping confidence intervals, all the data-poor compounds are not significantly different from each other. Finally, MultiFlow^®^ BMCs were evaluated and the lowest BMC of the clastogen biomarkers was selected as the point of departure (Figure 4C). This analysis revealed the following potency ranking: EPEG > DMBA > NSACB 7 > NSACB 3 > NSACB 4 > NSACB 2 > NSACB 9 > NSACB 5. As with TGx-DDI, two distinct groups can be observed for the data-poor compounds. 1) NSACB #7, 3, 4, and 2 make up one group with overlapping CIs, and 2) NSABC #9 and 5, also with overlapping CIs, make up a distinct, less potent, group. Overall, BMC modeling of the endpoints in the proposed *in vitro* testing strategy yielded nearly identical potency rankings for the genotoxic NSACB compounds.

### Integration of concentration-response data for a singular chemical prioritization strategy

**FIGURE 5:**
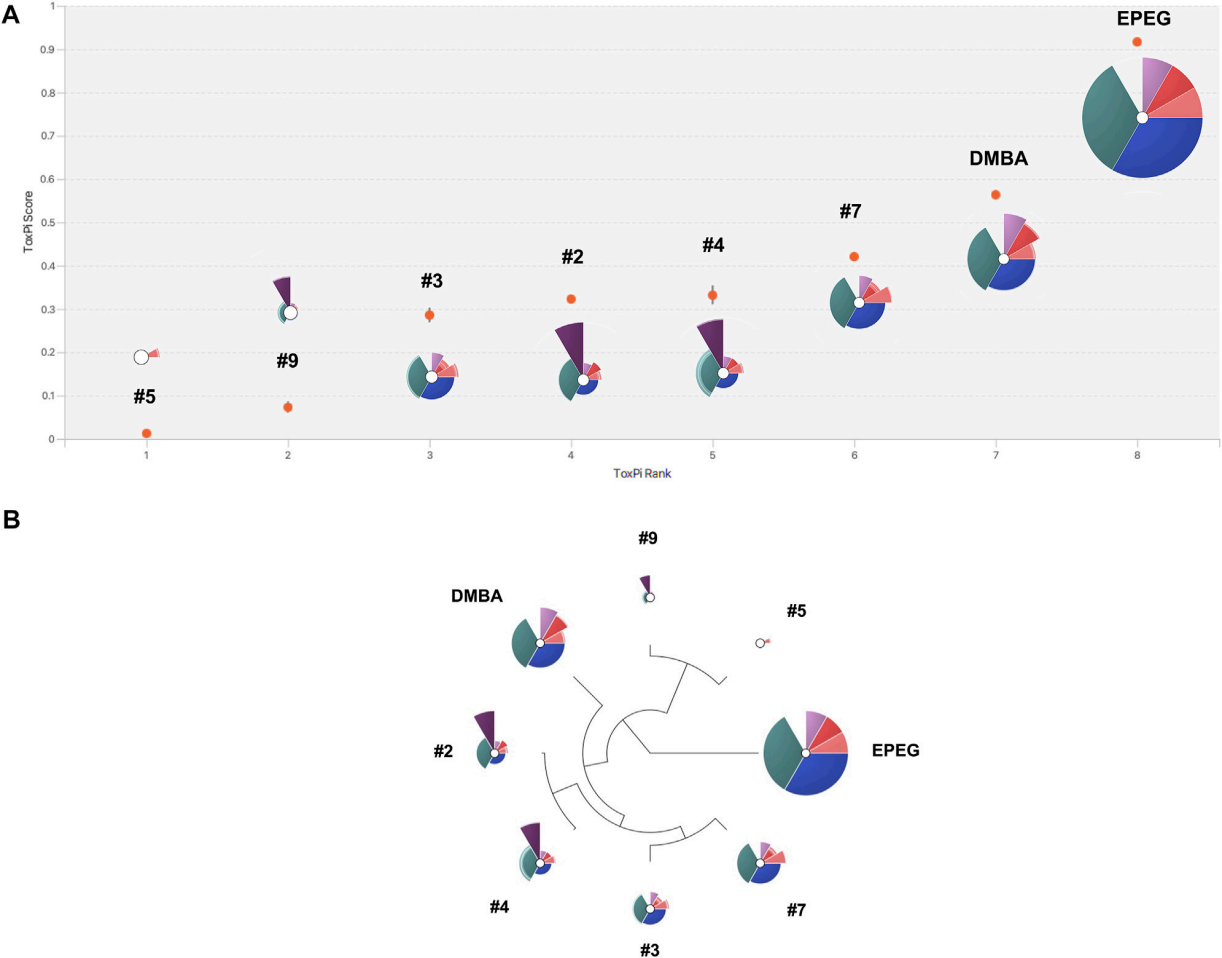
ToxPi visualization of multiplexed BMCs for the NSACB compounds with concordant positive hazard flags. **(A)** ToxPi score rankings and profiles for the data-poor compounds. For the ToxPi profiles, the distance of each slice from the origin indicates the slice score and endpoint potency (i.e., −log10 BMC). Slices represent the following endpoints: teal is TGx-DDI BMC, blue is MicroFlow^®^ BMC, pink and purple are the MultiFlow^®^ BMCs (i.e., dark pink is 24H p53, light pink is 4H p53, dark purple is 24H ɣH2AX, light purple is 4H ɣH2AX). Lower and upper bound confidence intervals are indicated by lighter shaded areas at the periphery of each slice. The width of each slice indicates the assigned endpoint weight. The TGx-DDI, MicroFlow^®^, and combined MultiFlow^®^ endpoints each represent 1/3 of the profile. **(B)** Hierarchical clustering of the ToxPi profiles. The ToxPi algorithm groups substances with similar toxicological profiles.

The ToxPi software was used to integrate all endpoint BMC metrics into a single score; the scores were subsequently used to rank the NSACB compounds and visualize mechanistic information regarding their genotoxic hazards (Figure 5; Supplementary Figure S7). With respect to the latter, the analyses generated toxicological profile graphics for each compound. The endpoints are represented by the coloured pie slices; the radius of each slice denotes the relative effect size expressed as −log10 BMC. Thus, a lower (i.e., more potent) BMC is indicated by greater protrusion from the origin. To compare to the independent endpoint BMC rankings displayed in Figure 4, the compounds with concordant positive hazard flags were first ranked based on their overall ToxPi scores for all endpoints (Figure 5A). The higher the overall ToxPi score, the more potent the compound. In this case, the most potent to least potent ranking was: EPEG > DMBA > NSACB 7 > NSACB 4 > NSACB 2 > NSACB 3 > NSACB 9 > NSACB 5. More specifically, based on the presented ToxPi profile (Figure 5A), EPEG is the most potent for five of the six endpoints examined: TGx-DDI, MN, 4H ɣH2AX, 24H p53 and 4H p53. In contrast, except for a small 4H p53 slice, NSACB #5 had almost no slices protruding from the center. The majority of the compounds, with the exception of NSACB #4, 2, and 9, lacked a 24H ɣH2AX slice. Comparisons of multiplexed ToxPi profiles (Figure 5B) showed that compounds #7, 3, 4, and 2 clustered together on a single branch; NSACB #9 and 5 clustered separately. This clustering is similar to the compound groupings observed in Figures 4A, C.

An additional ToxPi analysis was conducted for all NSACB compounds (Supplementary Figure S7), including those with discordant results (i.e., #1, 6, 8 and 10). In this case, the most potent to least potent ranking was: EPEG > DMBA > NSACB 7 > NSACB 4 > NSACB 1 > NSACB 2 > NSACB 3 > NSACB 8 > NSACB 9 > NSACB 6 > NSACB 5 > NSACB 10. The ToxPi profile of compound #1 clustered with #7, 3, 4 and 2, while the profiles of compounds #6, 8, and 10 clustered with #9 and 5.

## Discussion

TGx biomarkers are envisioned to provide a powerful NAM for modernizing toxicological testing by enabling rapid extraction of mechanistic information from data-rich transcriptomic datasets (Li et al., 2017). Preliminary data support that pairing TGx biomarkers, such as TGx-DDI, with additional markers of DNA damage (e.g., *in vitro* MN assay and high-throughput CometChip^®^) is a highly effective approach for accurate and efficient genotoxicity assessment (Buick et al., 2017; Buick et al., 2020; Buick et al., 2021). Flow-cytometry based assays have been developed to detect and quantify increases in markers of genotoxicity (i.e., MN, p53, ɣH2AX, p-H3, polyploidy). The integrated analysis of these endpoints has demonstrated efficacy in enhanced genotoxicity assessment (Smart et al., 2020; Avlasevich et al., 2021). Herein we combined the TGx-DDI transcriptomic biomarker in high-throughput format with the MicroFlow^®^ and MultiFlow^®^ DNA damage assays to explore application as an integrated test strategy for genotoxicity assessment of data-poor compounds. Human-relevant TK6 human lymphoblastoid cells were exposed to 10 data-poor test chemicals prioritized by Health Canada’s NSACB in conjunction with 3 control chemicals. We established hazard calls and potency ranking for each assay and then explored concordance and integration of the tests. Our results indicate that all ten NSACB compounds were positive in at least one assay. Six NSACB compounds (#2, 3, 4, 5, 7, and 9) had concordant results between all three assays, and four were discordant. NSACB #1 and #8 were positive in the TGx-DDI and MicroFlow^®^ assays, but were identified as non-genotoxicants by the MultiFlow^®^ assay. NSACB #6 and #10 tested positive only in the MicroFlow^®^ assay and were classified as non-DDI and non-genotoxicants by the TGx-DDI and MultiFlow^®^ assays. Though they provided critical insight individually, our work demonstrates the value of integrating these assays to strengthen confidence in hazard identification and chemical potency ranking.

To establish that the assays were performing as expected, the results of three reference control chemicals were examined. EPEG was identified as positive by the TGx-DDI biomarker, had significant concentration-dependent fold-increases in MN, and induced the clastogen biomarkers 4H/24H p53 and 4H ɣH2AX. As a topoisomerase II inhibitor, EPEG inhibits DNA synthesis and causes double-strand breaks (Montecucco et al., 2015). Similarly, the positive control chemical DMBA displayed a strong TGx-DDI response, MN induction, and increases in the clastogen biomarkers 4H and 24H ɣH2AX. DMBA is metabolized into 3,4-diol-1,2-epoxide that reacts with DNA to form bulky adducts (RamaKrishna et al., 1992); DMBA has been shown to induce chromosome damage *in vitro* (Matsushima et al., 1999; Von Der Hude et al., 2000) and *in vivo* (De Boeck et al., 2005). In contrast, the negative control, DMANN, did not yield any positive calls in any of the assays. It is well established that DMANN is non-genotoxic; and it is a negative control for assessing the performance of new or improved genotoxicity tests (Kirkland et al., 2016). Thus, the results obtained by the test strategy were in line with expectations based on the positive and negative controls included in the study.

We then explored the hazard calls made for the data-poor NSACB compounds. Eight out of ten (NSACB #1, 2, 3, 4, 5, 7, 8, and 9) were classified as DDI; all ten were positive for MN induction; five were identified as clastogens (NSACB #2, 3, 5, 7, 9); and one was identified as a pan-genotoxicant (NSACB #4) in at least one S9 condition. Six of our data-poor compounds (NSACB #2, 3, 4, 5, 7, and 9) had concordant results; i.e., DDI, MN inducing, and clastogenic calls for the TGx-DDI, MicroFlow^®^, MultiFlow^®^ assays, respectively. Thus, we conclude that these compounds are genotoxic *in vitro*, causing DNA damage *via* a clastogenic MoA. Although these are data-poor compounds, there are a few supporting reports for these findings. For example, NSACB #7 caused a statistically significant and biologically relevant increase in the frequency of %MN with S9 (OECD 487) and an equivocal result without S9 (SCCP, 2006). Similarly, we observed a stronger %MN frequency in the +S9 condition at the highest concentration (Supplemental Figure S4). NSACB #5 and 9 were positive in previous studies using the *in vitro* mammalian chromosomal aberration test (OECD 473) (SCCP, 2007; SCCS, 2013). NSACB #5 was clastogenic following a short-term (6H) treatment with S9 and after continuous treatment (24H) without S9 (SCCS, 2013). This aligns with our MultiFlow^®^ results showing a strong response in clastogen-related biomarkers in both S9 conditions for multiple concentrations (i.e., 4H ɣH2AX, 4H p53). NSACB #9 induced structural chromosomal aberrations in the presence of S9 and the results were equivocal without S9 (SCCP, 2007). In our MultiFlow^®^ results, we observed strong fold-increases in clastogenic biomarkers (i.e., 4H and 24H ɣH2AX) in both S9 conditions. Overall, our results contribute to the weight-of-evidence for the genotoxicity of these compounds and provide new evidence for those where little toxicological data, if any, exist.

Conversely, there were four instances of discordant test results across the three assays. Two compounds, NSACB #6 and #10, were identified as non-DDI by TGx-DDI but yielded positive results in the MN test (NSACB #10 + S9 only, NSACB #6 −S9 and +S9). Since MN can result from mechanisms affecting cell division/mitotic machinery leading to aneuploidy (i.e., aneugenicity) (Luzhna et al., 2013), we speculated that these compounds may be aneugens. An example of this is colchicine, a known aneugenic agent, which is positive for MN but negative (non-DDI) by TGx-DDI (Buick et al., 2020). An advantage of integrating the MultiFlow^®^ assay into the test strategy is that it can differentiate clastogenic from aneugenic mechanisms. The MultiFlow^®^ assay classified both NSACB #6 and #10 as non-genotoxicants. Thus, the MultiFlow^®^ results suggest that NSACB #6 and #10 are likely irrelevant positives in the MN test. It is well established that genotoxicity tests in mammalian cells have low specificity in predicting genotoxic effects manifested *in vivo*, often reporting irrelevant positives that do not pose an appreciable mutagenic risk in humans (Kirkland et al., 2007; Guyton et al., 2009; Dearfield et al., 2011). Indeed, Kirkland et al. (2005), Kirkland et al. (2006) determined that mammalian *in vitro* chromosome damage tests in particular had specificity for rodent carcinogenicity of <45%. This could be due to several factors. For example, at high concentrations some chemical agents induce mammalian cell death, and certain types of cell death (e.g., apoptosis) cause nuclear condensation and fragmentation as part of this process. These nuclear fragments can be detected as micronuclei, even though they were formed as a result of cellular metabolism, and not as a direct DNA-damaging mechanism induced by chemical agents (Williams et al., 1974). Apoptosis is a known confounder for the micronucleus assay that can produce an irrelevant positive *in vitro* genotoxicity call (Avlasevich et al., 2021). Known apoptogens such as carbonyl cyanide m-chlorphenyl hydrazone (CCCP), brefeldin A, and thapsigargin, have been shown to induce MN in the MicroFlow^®^ assay, but were correctly classified as non-genotoxicants by a follow-up MultiFlow^®^ assay; thus, resulting in an improved assay specificity (Avlasevich et al., 2021). Moreover, the TGx-DDI transcriptomic biomarker was originally designed to provide biological relevance as a complement to positive chromosome damage assay results by identifying changes in gene expression that predict a DDI mechanism (Li et al., 2017). In subsequent works, Li et al. (2017) have demonstrated the utility of the biomarker for this purpose. When ten known irrelevant positive compounds that were positive in the *in vitro* chromosome damage assay were tested with the TGx-DDI biomarker, TGx-DDI correctly identified 9 out of 10 compounds as negative. Therefore, it is plausible from our results that NSACB #6 and #10 are irrelevant positives inducing *in vitro* positive MN as a result of apoptosis.

The last two compounds, NSACB #1 and #8, were identified as weakly DDI by TGx-DDI (both in +S9 conditions only) and MN-inducing by MicroFlow^®^ (#1 in both S9 conditions, #8 only +S9). However, they were both also identified as non-genotoxicants by the MultiFlow^®^ assay. Given that both the TGx-DDI biomarker and MultiFlow^®^ assay are DNA damage reporter assays that rely on transcriptional and cellular changes, respectively, in DNA damage response pathways (i.e., p53 and related genes), we expected to see a high correlation in hazard calls between these two assays. Of the 13 test chemicals, 11 (i.e., 85%) had concordant calls between these two assays; NSACB #1 and #8 are the only two where this was not the case. However, it is important to note the weakness of the DDI calls for these two compounds. NSACB #1 had one DDI call at the highest concentration in the NSC-PA only. NSACB #8 had two DDI calls at the highest concentration by NSC-PA and PCA. In addition, the PCA call was visibly borderline, with the concentration grouping with the training set DDI compounds just left of the classification line on the PCA plot (Supplemental Figure S3K). Moreover, in both cases the highest concentration analyzed was very close to the viability threshold of 40% (i.e., 43% for NSACB #1 +S9, and 46% for NSACB #8); thus, high cytotoxicity could be leading to a misclassification. Alternatively, the discordant results between TGx-DDI and MultiFlow^®^ could be due to different assay sensitivities. Non-etheless, both compounds induced strong concentration-dependent increases in %MN. Therefore, although these results are somewhat ambiguous, in order to be conservative further analyses should be considered to explore their DDI potential. This could include mutagenicity testing *in vitro*; alternatively, their transcriptomic profiles could be examined for further mechanistic insights. One advantage of using the TempO-Seq^TM^ assay for the TGx-DDI classification is that the transcriptomic dataset is not limited to the 64 biomarker genes; there are a total of 2,730 genes in the TempO-Seq™ S1500 + set (Mav et al., 2018). Thus, applying high-throughput transcriptomic test strategies, such as the one developed by Harrill et al. (2021), could also be used to identify biological perturbations *via* pathway analyses and derive transcriptional biological pathway altering concentrations (BPACs) to provide more insight into the toxicity of NSACB compounds #1 and #8.

In addition to determining qualitative genotoxicity calls (i.e., hazard identification), there has been a growing momentum in quantitative analyses of genotoxicity concentration-response data to derive potency metrics for potency ranking and regulatory decision-making (Johnson et al., 2014; MacGregor et al., 2015; White et al., 2020). BMC modeling aims to determine the concentration required to elicit a predefined change in response in relation to background. Herein, we investigated the consistency of potency rankings based on BMC analysis across these *in vitro* assays. In order to directly compare the rankings we limited our analysis to the six compounds and two controls that were positive across all three assays. First, we derived an overall BMC for the TGx-DDI biomarker by calculating a bootstrap BMC confidence interval. Previous work has established that transcriptomic BMD values correlate well with those from apical endpoints *in vivo* (Thomas et al., 2011; Thomas et al., 2013; Farmahin et al., 2017). Moreover, as shown by Buick et al. (2021), the bootstrap median BMC enables more biomarker genes to be modeled, allows for the generation of 95% confidence intervals, and resulted in identical potency rankings between TGx-DDI and the *in vitro* high-throughput comet assay for six compounds. Second, to calculate an overall MultiFlow^®^ BMC, the four clastogen biomarkers (i.e., 4H/24H p53, 4H/24H ɣH2AX) were modeled for each compound. The biomarker with the lowest BMC was selected as the BMC for potency ranking since this would represent the most sensitive, and thus, conservative, endpoint. Using this approach, the potency ranking for all the assays were nearly identical. The only exception was NSACB #3, which was slightly more potent in the TGx-DDI assay, surpassing NSACB #4 and #2. However, the confidence intervals overlapped for these three compounds and thus they were not significantly different from each other. Two main groupings (i.e., NSACB #7, 4, 2, 3 and NSACB # 9, 5) were observed for both the TGx-DDI and MultiFlow^®^ chemical rankings. For MicroFlow^®^, the confidence intervals were larger resulting in one group encompassing all data-poor compounds that were not significantly different from each other. Due to the differences in confidence interval ratios, the TGx-DDI and MultiFlow^®^ assays provided better potency discrimination compared to the MicroFlow^®^ assay. Overall, independent quantitative analyses derived concordant potency rankings and groupings across the assays.

It should be noted that the quantitative metrics (i.e., BMCs) generated in this study can be converted into administered equivalent doses (AEDs) by applying *in vitro* to *in vivo* extrapolation (IVIVE) using high-throughput toxicokinetic (HTTK) models. AED values represent the estimated oral dose required to generate a steady state concentration in the plasma (i.e., *in vivo*) that is equivalent to the genotoxic concentration *in vitro*. The values can be employed to calculate margin of exposure values (e.g., bioactivity exposure ratios (BERs)) that can be used in risk assessment (Kuo et al., 2022). In this study, a different benchmark response (BMR) was applied to each assay making it difficult to directly compare the BMC values derived for each compound in order to select one for IVIVE modeling. Generally, the BMC values derived from the TGx-DDI and MicroFlow^®^ assays were similar and markedly more conservative than those derived from the MultiFlow^®^ assay. Thus, for each compound, AEDs could be determined for all BMCs and used to calculate and display a range of BER values. Although, as the compounds studied are data-poor, it may be challenging to derive AEDs and BERs in this case. HTTK data would likely need to be generated from *in vitro* plasma protein binding and metabolic clearance assays to predict *in vivo* effects (Judson et al., 2014). Moreover, it is not clear if there would be enough exposure information on these compounds to derive BERs for risk assessment activities.

As modernized *in vitro* test strategies are being developed and applied for qualitative and quantitative analysis there is a growing need to supplement this work with new approaches that combine and interpret the large amounts of data generated for decision making. Prioritization software tools, such as ToxPi, that combine results from multiple data streams and reduce it to one metric have been used for this purpose. Avlasevich et al. (2021) explored the integration of quantitative BMC modeling with the ToxPi software to combine and visualize concentration-response data from MultiFlow^®^ and MicroFlow^®^ endpoints to derive a unitless ToxPi score for a singular chemical prioritization strategy. We applied a similar approach to our genotoxicity assessment by aggregating all endpoint BMC metrics into ToxPi scores to rank the NSACB compounds and visualize their genotoxic hazards. To adequately compare to the individual assay potency rankings, only the concordant positive compounds were modeled (Figure 5). Remarkably, we observed a highly similar ToxPi pattern to the individual assay potency rankings. EPEG and DMBA were the most potent and the NSACB compound rankings were identical to the TGx-DDI assay ranking. It is important to note that we eliminated the 24H ɣH2AX endpoint for many compounds. This is due to a lack of a robust response in this endpoint resulting in an incalculable BMC or infinite BMCU; thus, for a meaningful ToxPi score comparison it is important to have a set of chemicals that elicit robust responses for each endpoint.

It is important to highlight one major caveat with the ToxPi approach. For each analysis, the ToxPi scores are derived as a relative comparison of the compounds in the dataset. Thus, each score is only meaningful in the context in which it was produced (USNRC, 2014). Consequently, comparing ToxPi scores from separate analyses with different sets of compounds would be misleading; a new analysis would need to be completed with all the compounds in question to accurately compare their potencies.

For the test strategy investigated in this study to be applied for further screening of data-poor compounds, it will first be essential to establish scientific confidence in this NAM-based approach for regulatory acceptance. Increasing numbers of new testing methods are being developed to improve chemical (geno) toxicological assessment; however, there is a growing bottleneck when it comes to implementation of these methods for risk assessment activities. Traditional validation processes, including the OECD Guidance Document (OECD GD 34) on the Validation and International Acceptance of New or Updated Test Methods for Hazard Assessment (OECD, 2005) can be time-consuming and complicated to implement. Thus, there is growing recognition that updated frameworks, such as the ones described by van der Zalm et al. (2022) and Parish et al. (2020), are urgently needed to increase confidence in NAMs, thereby supporting accelerated regulatory uptake that is nevertheless aligned with the key principles employed for traditional validations (Parish et al., 2020; van der Zalm et al., 2022). As such, in this case study we have defined and demonstrated the context-of-use for the proposed integrated NAM-based test strategy described herein as the screening of data-poor compounds for genotoxicity hazard identification and prioritization. Future work will need to conduct technical characterization to assess the performance (i.e., accuracy) and reproducibility of the multiplexed NAM, as well as to establish human relevance of the endpoints examined [e.g., endpoints relevant to adverse outcome pathways (AOPs)]. Performance studies are underway within the GeneTox21 research program to evaluate the proposed integrated NAM-based test strategy (in addition to three other modernized *in vitro* assays) with four classes of reference compounds with diverse mechanisms.

In summary, this study demonstrates that the integration of an established transcriptomic assay (i.e., TGx-DDI transcriptomic biomarker) and two flow cytometry-based assays (MultiFlow^®^ DNA Damage Assay and *in vitro* MicroFlow^®^) enabled an effective *in vitro*-only assessment of genotoxicity and revealed detailed mechanistic insights for ten data-poor compounds that were prioritized for evaluation by *in silico* screening. The considerable genotoxicity data generated in this study will provide regulators with additional information regarding the hazard of these data-poor NSACB compounds. Moreover, comparison of BMC values derived from modeling concentration-response data enabled potency ranking of these compounds for further prioritization. Ultimately, this work applies a modernized NAM-based approach for effective genotoxicity assessment, including chemical prioritization for further regulatory scrutiny. Importantly, the result of this work can be used to assess these assays and the proposed test strategy by applying a NAMs confidence framework, a critical step in order to advance adoption and implementation of NAMs for chemical risk assessment.

## Data Availability

The datasets presented in this study can be found in online repositories. The names of the repository/repositories and accession number(s) can be found below: https://www.ncbi.nlm.nih.gov/geo/, GSE213454.
