# Supplementary material for: Application of a new approach methodology (NAM)-based strategy for genotoxicity assessment of data-poor compounds
Source: Front Toxicol. 2023 Jan 23;5:1098432. doi: 10.3389/ftox.2023.1098432 (PMC9899896; doi:10.3389/ftox.2023.1098432)
Supplement: Supplementary file 1 [file DataSheet1.DOCX]

Supplementary Material

# Supplementary Figures

**
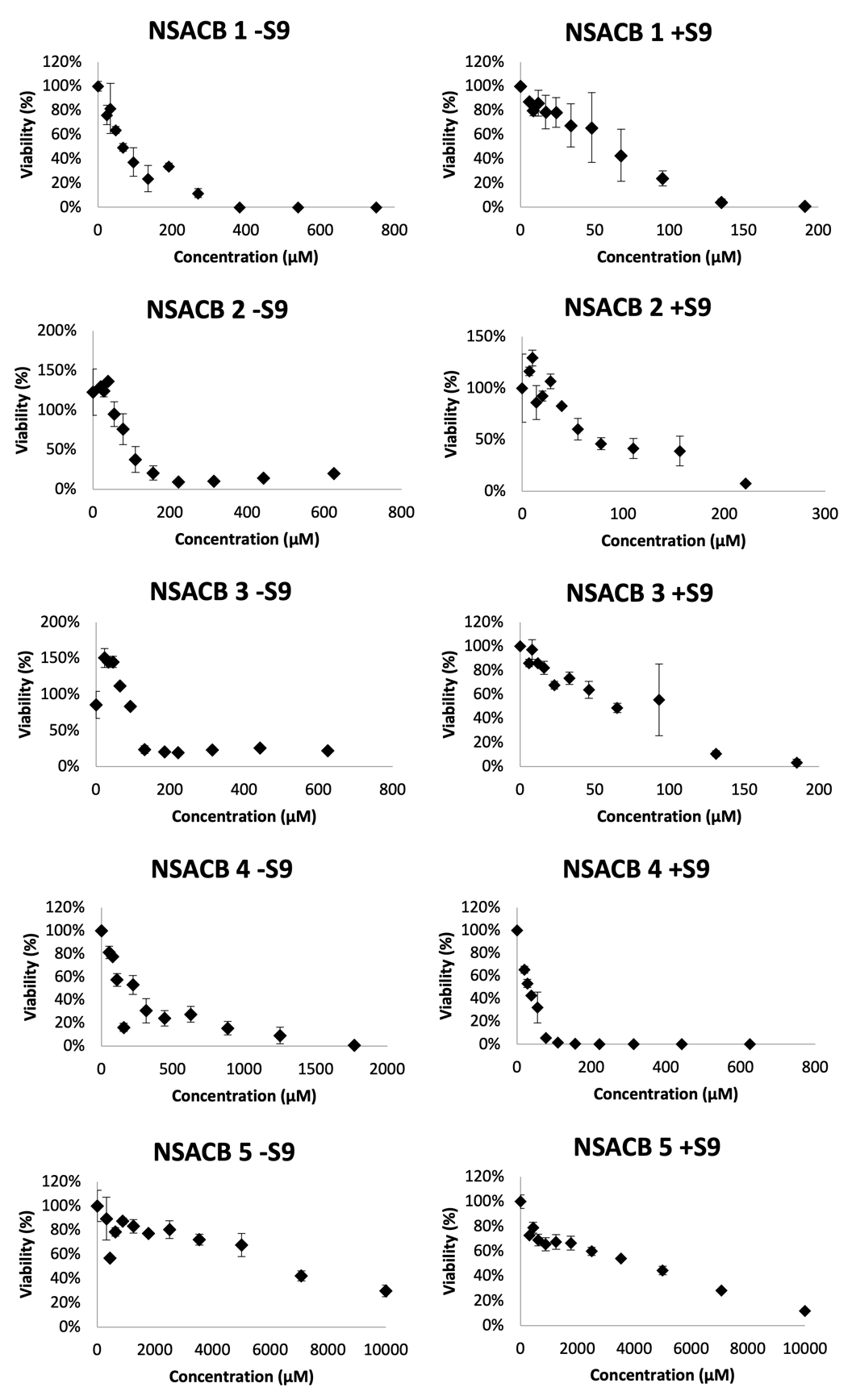
**


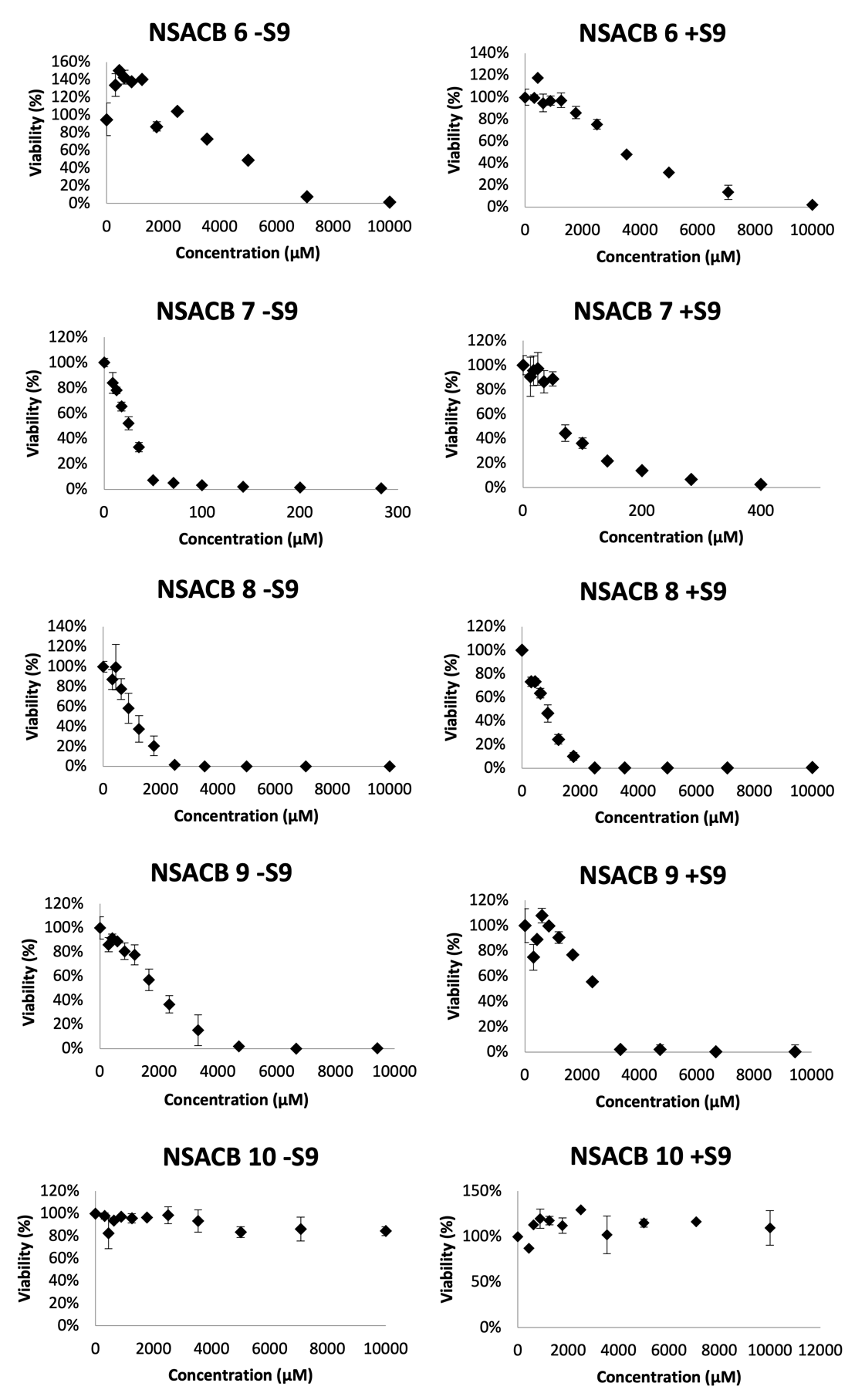


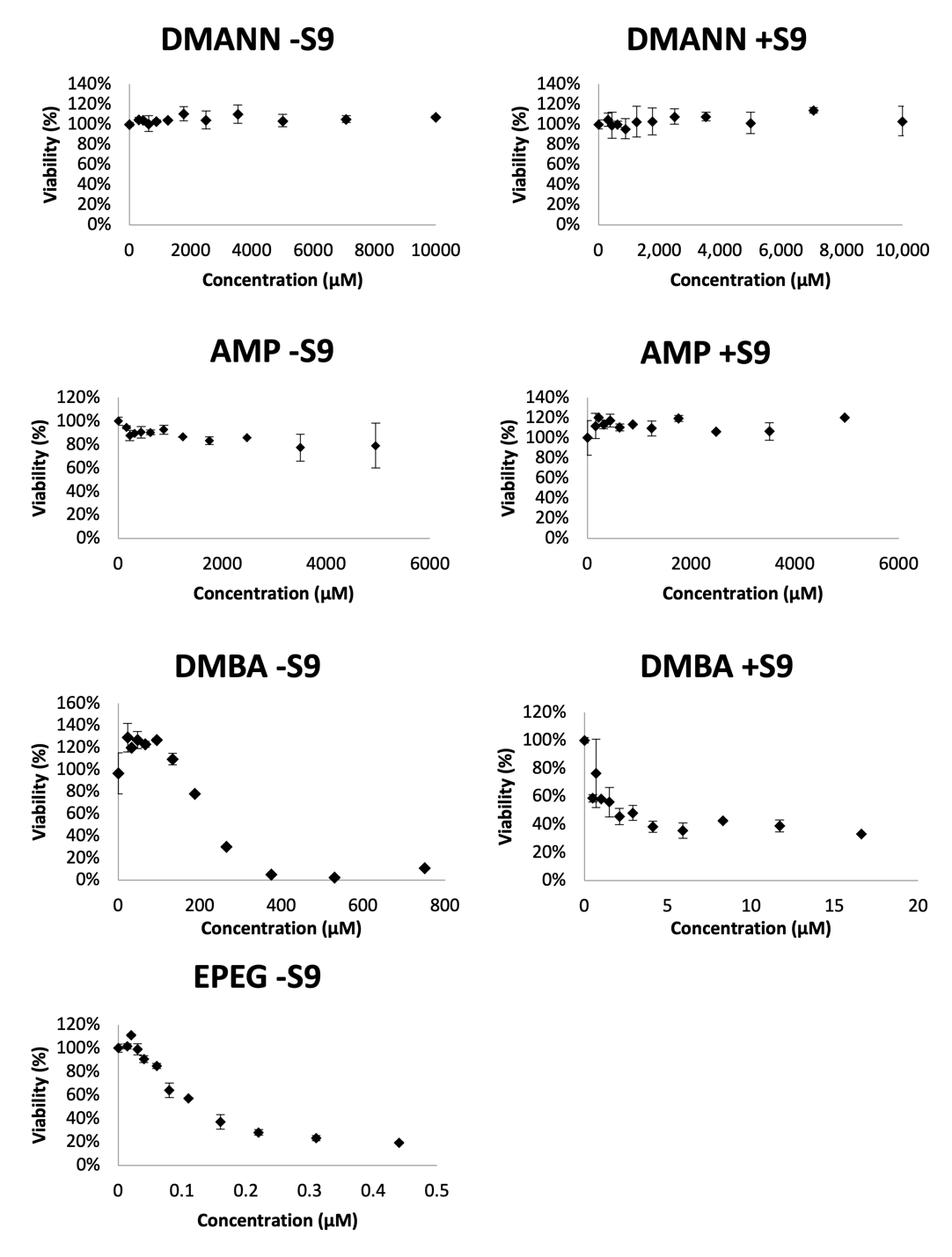


**Supplementary Figure 1: Viability results for the test chemicals used in this study.** All chemicals (with the exception of EPEG) were tested with (+S9) and without (-S9) metabolic activation.


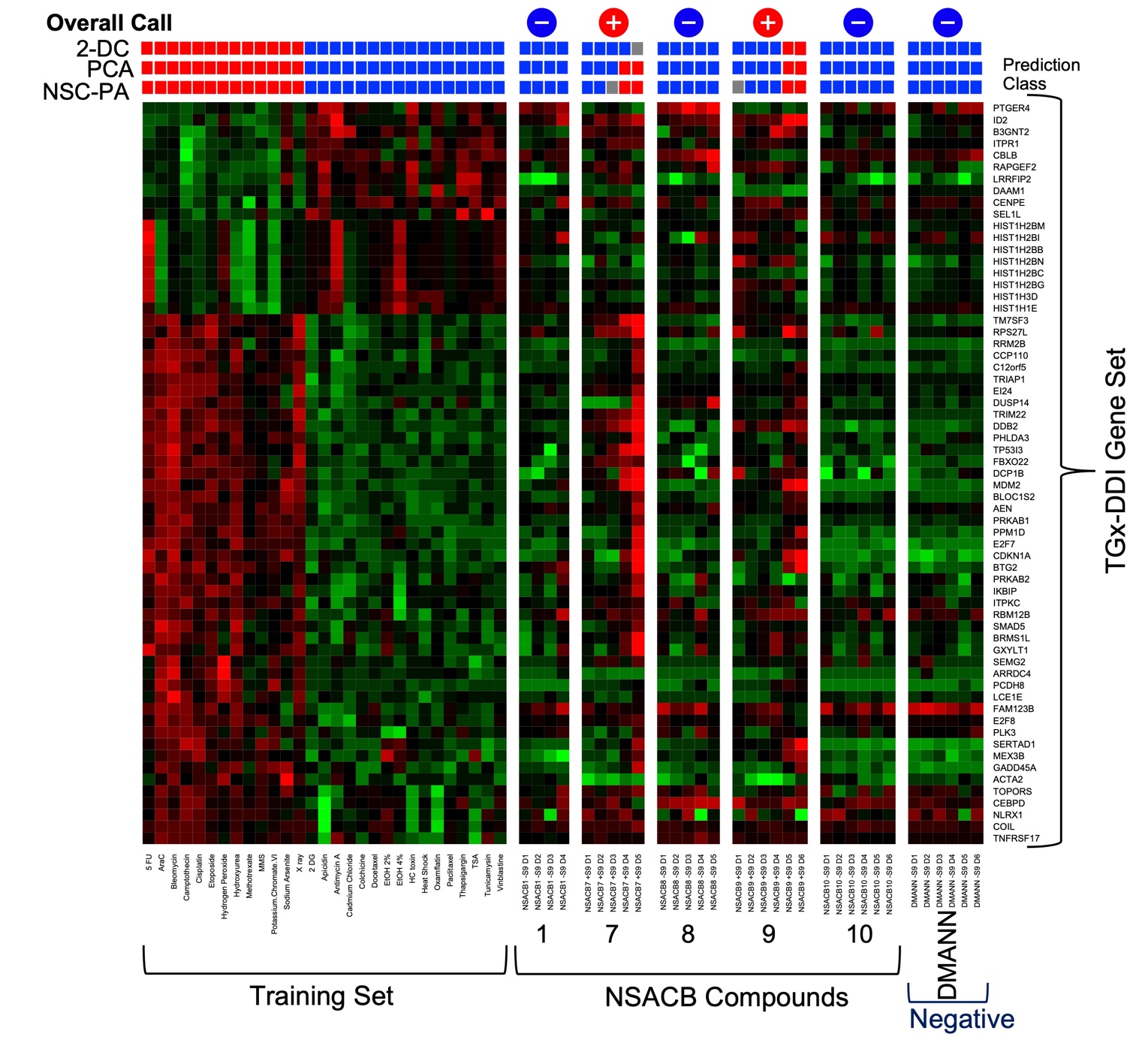


**Supplementary Figure 2: TGx-DDI classification of NSACB data-poor compounds.** The heatmap on the left depicts the 28 reference chemicals used as a training set to generate the biomarker. The color scale indicates the average gene expression fold changes of two replicates relative to solvent control: up-regulated genes are shown in red, down-regulated genes are shown in green, genes with no change are shown in black. Three analyses: (1) 2-dimensional hierarchical clustering (2-DC), (2) principal component analysis (PCA), and (3) nearest shrunken centroid probability analysis (NSC-PA) were used to determine classification probabilities shown for all treatment conditions using red (genotoxic), blue (non-genotoxic), and grey (inconclusive) boxes. The overall calls are also shown at the top of each column: “+” signifies a positive DDI call, “-” signifies a non-DDI call. D1 represents the lowest concentration tested, D6 the highest. Cytotoxic concentrations (< 40% relative survival) were removed from the analysis. Presented -S9 condition for #1, 8, 10, DMANN. Presented +S9 condition for #7 and 9.

**Supplementary Figure 3A: NASCB 1 (-S9) TGx-DDI biomarker classification using the Principal Component Analysis (PCA) (left) and hierarchical clustering (right) statistical analyses.** Genotoxic reference chemicals are shown in red text, non-genotoxic reference chemicals are shown in blue text, and the test agent is shown in the green text. The line drawn on the PCA plot and the main branch on the dendogram divides the genotoxic and non-genotoxic agents and was used to classify the test compounds. D1 represents the lowest concentration tested, D4 the highest.

**Supplementary Figure 3B: NASCB 1 (+S9) TGx-DDI biomarker classification using the Principal Component Analysis (PCA) (left) and hierarchical clustering (right) statistical analyses.** Genotoxic reference chemicals are shown in red text, non-genotoxic reference chemicals are shown in blue text, and the test agent is shown in the green text. The line drawn on the PCA plot and the main branch on the dendogram divides the genotoxic and non-genotoxic agents and was used to classify the test compounds. D1 represents the lowest concentration tested, D5 the highest.

**Supplementary Figure 3C: NASCB 2 (+S9) TGx-DDI biomarker classification using the Principal Component Analysis (PCA) (left) and hierarchical clustering (right) statistical analyses.** Genotoxic reference chemicals are shown in red text, non-genotoxic reference chemicals are shown in blue text, and the test agent is shown in the green text. The line drawn on the PCA plot and the main branch on the dendogram divides the genotoxic and non-genotoxic agents and was used to classify the test compounds. D1 represents the lowest concentration tested, D5 the highest.

**Supplementary Figure 3D: NASCB 3 (+S9) TGx-DDI biomarker classification using the Principal Component Analysis (PCA) (left) and hierarchical clustering (right) statistical analyses.** Genotoxic reference chemicals are shown in red text, non-genotoxic reference chemicals are shown in blue text, and the test agent is shown in the green text. The line drawn on the PCA plot and the main branch on the dendogram divides the genotoxic and non-genotoxic agents and was used to classify the test compounds. D1 represents the lowest concentration tested, D6 the highest.

**Supplementary Figure 3E: NASCB 4 (+S9) TGx-DDI biomarker classification using the Principal Component Analysis (PCA) (left) and hierarchical clustering (right) statistical analyses.** Genotoxic reference chemicals are shown in red text, non-genotoxic reference chemicals are shown in blue text, and the test agent is shown in the green text. The line drawn on the PCA plot and the main branch on the dendogram divides the genotoxic and non-genotoxic agents and was used to classify the test compounds. D1 represents the lowest concentration tested, D5 the highest.

**Supplementary Figure 3F: NASCB 5 (+S9) TGx-DDI biomarker classification using the Principal Component Analysis (PCA) (left) and hierarchical clustering (right) statistical analyses.** Genotoxic reference chemicals are shown in red text, non-genotoxic reference chemicals are shown in blue text, and the test agent is shown in the green text. The line drawn on the PCA plot and the main branch on the dendogram divides the genotoxic and non-genotoxic agents and was used to classify the test compounds. D1 represents the lowest concentration tested, D6 the highest.

**Supplementary Figure 3G: NASCB 6 (+S9) TGx-DDI biomarker classification using the Principal Component Analysis (PCA) (left) and hierarchical clustering (right) statistical analyses.** Genotoxic reference chemicals are shown in red text, non-genotoxic reference chemicals are shown in blue text, and the test agent is shown in the green text. The line drawn on the PCA plot and the main branch on the dendogram divides the genotoxic and non-genotoxic agents and was used to classify the test compounds. D1 represents the lowest concentration tested, D5 the highest.

**Supplementary Figure 3H: NASCB 7 (-S9) TGx-DDI biomarker classification using the Principal Component Analysis (PCA) (left) and hierarchical clustering (right) statistical analyses.** Genotoxic reference chemicals are shown in red text, non-genotoxic reference chemicals are shown in blue text, and the test agent is shown in the green text. The line drawn on the PCA plot and the main branch on the dendogram divides the genotoxic and non-genotoxic agents and was used to classify the test compounds. D1 represents the lowest concentration tested, D5 the highest.

**Supplementary Figure 3I: NASCB 7 (+S9) TGx-DDI biomarker classification using the Principal Component Analysis (PCA) (left) and hierarchical clustering (right) statistical analyses.** Genotoxic reference chemicals are shown in red text, non-genotoxic reference chemicals are shown in blue text, and the test agent is shown in the green text. The line drawn on the PCA plot and the main branch on the dendogram divides the genotoxic and non-genotoxic agents and was used to classify the test compounds. D1 represents the lowest concentration tested, D5 the highest.

**Supplementary Figure 3J: NASCB 8 (-S9) TGx-DDI biomarker classification using the Principal Component Analysis (PCA) (left) and hierarchical clustering (right) statistical analyses.** Genotoxic reference chemicals are shown in red text, non-genotoxic reference chemicals are shown in blue text, and the test agent is shown in the green text. The line drawn on the PCA plot and the main branch on the dendogram divides the genotoxic and non-genotoxic agents and was used to classify the test compounds. D1 represents the lowest concentration tested, D5 the highest.

**Supplementary Figure 3K: NASCB 8 (+S9) TGx-DDI biomarker classification using the Principal Component Analysis (PCA) (left) and hierarchical clustering (right) statistical analyses.** Genotoxic reference chemicals are shown in red text, non-genotoxic reference chemicals are shown in blue text, and the test agent is shown in the green text. The line drawn on the PCA plot and the main branch on the dendogram divides the genotoxic and non-genotoxic agents and was used to classify the test compounds. D1 represents the lowest concentration tested, D5 the highest.

**Supplementary Figure 3L: NASCB 9 (-S9) TGx-DDI biomarker classification using the Principal Component Analysis (PCA) (left) and hierarchical clustering (right) statistical analyses.** Genotoxic reference chemicals are shown in red text, non-genotoxic reference chemicals are shown in blue text, and the test agent is shown in the green text. The line drawn on the PCA plot and the main branch on the dendogram divides the genotoxic and non-genotoxic agents and was used to classify the test compounds. D1 represents the lowest concentration tested, D5 the highest.

**Supplementary Figure 3M: NASCB 9 (+S9) TGx-DDI biomarker classification using the Principal Component Analysis (PCA) (left) and hierarchical clustering (right) statistical analyses.** Genotoxic reference chemicals are shown in red text, non-genotoxic reference chemicals are shown in blue text, and the test agent is shown in the green text. The line drawn on the PCA plot and the main branch on the dendogram divides the genotoxic and non-genotoxic agents and was used to classify the test compounds. D1 represents the lowest concentration tested, D6 the highest.

**Supplementary Figure 3N: NASCB 10 (-S9) TGx-DDI biomarker classification using the Principal Component Analysis (PCA) (left) and hierarchical clustering (right) statistical analyses.** Genotoxic reference chemicals are shown in red text, non-genotoxic reference chemicals are shown in blue text, and the test agent is shown in the green text. The line drawn on the PCA plot and the main branch on the dendogram divides the genotoxic and non-genotoxic agents and was used to classify the test compounds. D1 represents the lowest concentration tested, D6 the highest.

**Supplementary Figure 3O: NASCB 10 (+S9) TGx-DDI biomarker classification using the Principal Component Analysis (PCA) (left) and hierarchical clustering (right) statistical analyses.** Genotoxic reference chemicals are shown in red text, non-genotoxic reference chemicals are shown in blue text, and the test agent is shown in the green text. The line drawn on the PCA plot and the main branch on the dendogram divides the genotoxic and non-genotoxic agents and was used to classify the test compounds. D1 represents the lowest concentration tested, D6 the highest.

**Supplementary Figure 3P: EPEG (-S9) TGx-DDI biomarker classification using the Principal Component Analysis (PCA) (left) and hierarchical clustering (right) statistical analyses.** Genotoxic reference chemicals are shown in red text, non-genotoxic reference chemicals are shown in blue text, and the test agent is shown in the green text. The line drawn on the PCA plot and the main branch on the dendogram divides the genotoxic and non-genotoxic agents and was used to classify the test compounds. D1 represents the lowest concentration tested, D5 the highest.

**Supplementary Figure 3Q: DMBA (+S9) TGx-DDI biomarker classification using the Principal Component Analysis (PCA) (left) and hierarchical clustering (right) statistical analyses.** Genotoxic reference chemicals are shown in red text, non-genotoxic reference chemicals are shown in blue text, and the test agent is shown in the green text. The line drawn on the PCA plot and the main branch on the dendogram divides the genotoxic and non-genotoxic agents and was used to classify the test compounds. D1 represents the lowest concentration tested, D5 the highest.

**Supplementary Figure 3R: DMANN (-S9) TGx-DDI biomarker classification using the Principal Component Analysis (PCA) (left) and hierarchical clustering (right) statistical analyses.** Genotoxic reference chemicals are shown in red text, non-genotoxic reference chemicals are shown in blue text, and the test agent is shown in the green text. The line drawn on the PCA plot and the main branch on the dendogram divides the genotoxic and non-genotoxic agents and was used to classify the test compounds. D1 represents the lowest concentration tested, D6 the highest.

**Supplementary Figure 3S: DMANN (+S9) TGx-DDI biomarker classification using the Principal Component Analysis (PCA) (left) and hierarchical clustering (right) statistical analyses.** Genotoxic reference chemicals are shown in red text, non-genotoxic reference chemicals are shown in blue text, and the test agent is shown in the green text. The line drawn on the PCA plot and the main branch on the dendogram divides the genotoxic and non-genotoxic agents and was used to classify the test compounds. D1 represents the lowest concentration tested, D6 the highest.


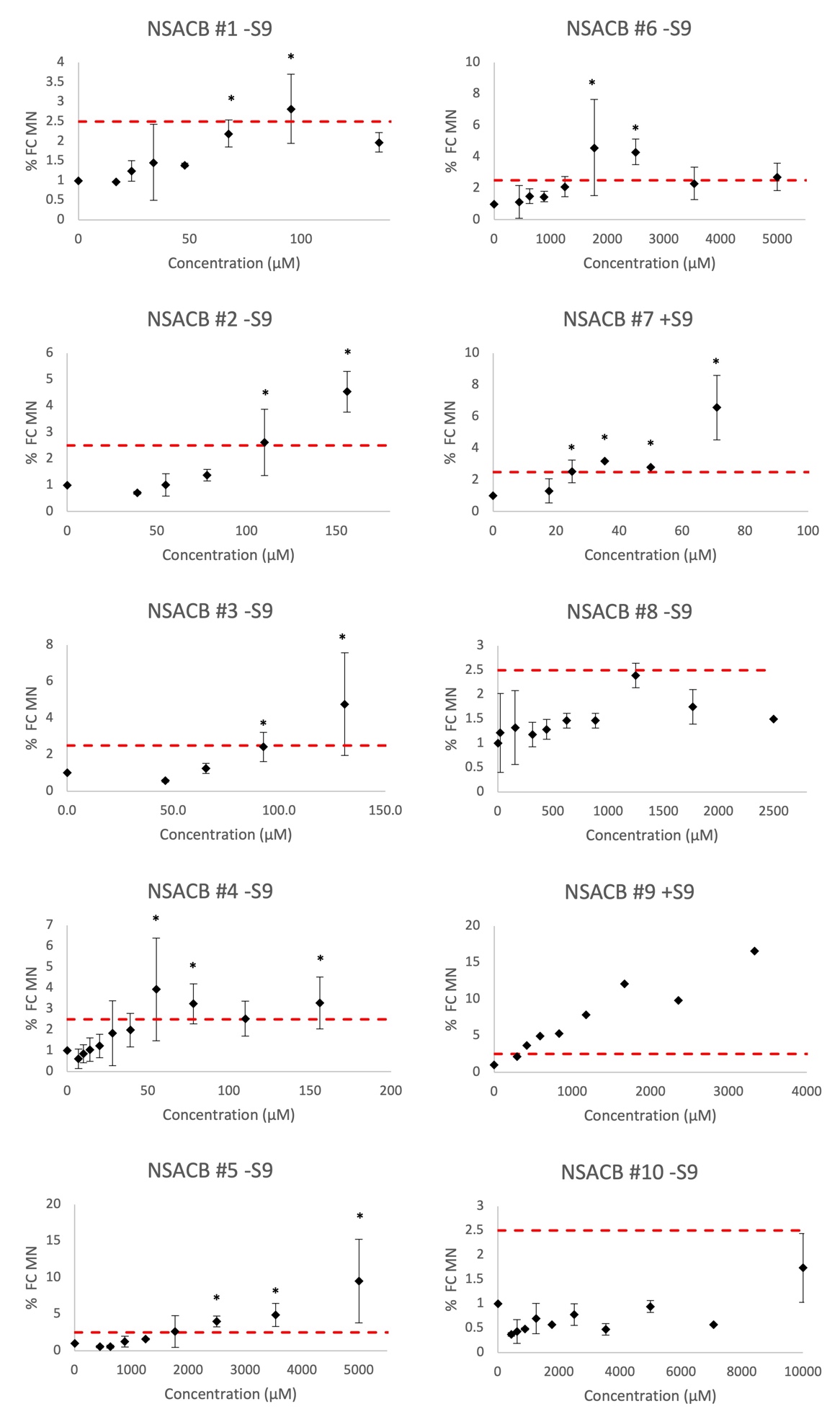


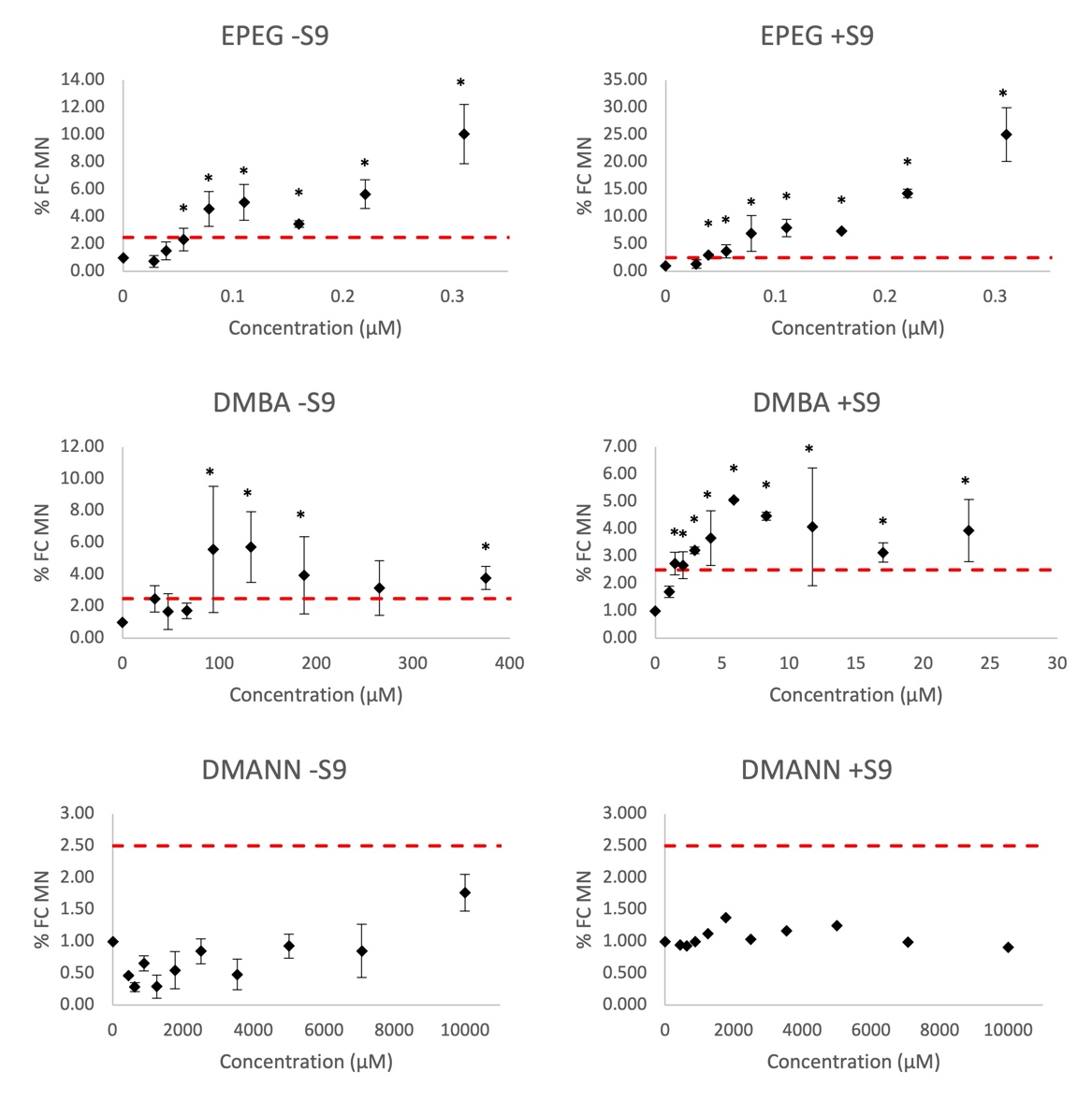


**Supplementary Figure 4: In vitro MicroFlow® assay results for ten NSACB data-poor substances (#1-10) and control chemicals.** Fold-change in % micronucleus (% FC MN) compared to vehicle control is depicted by black diamonds. The dashed red line shows the threshold (i.e., 2.5-fold increase in %MN) required to yield a positive classification. Statistically significant (p < 0.05) increases in %MN in comparison with the concurrent vehicle control are designated by an asterisk (*). Cytotoxic concentrations (< 40% viability) were removed from the analysis. Error bars denote standard deviation from mean. N=2 except for NSACB #9 +S9 and DMANN +S9 where N=1.


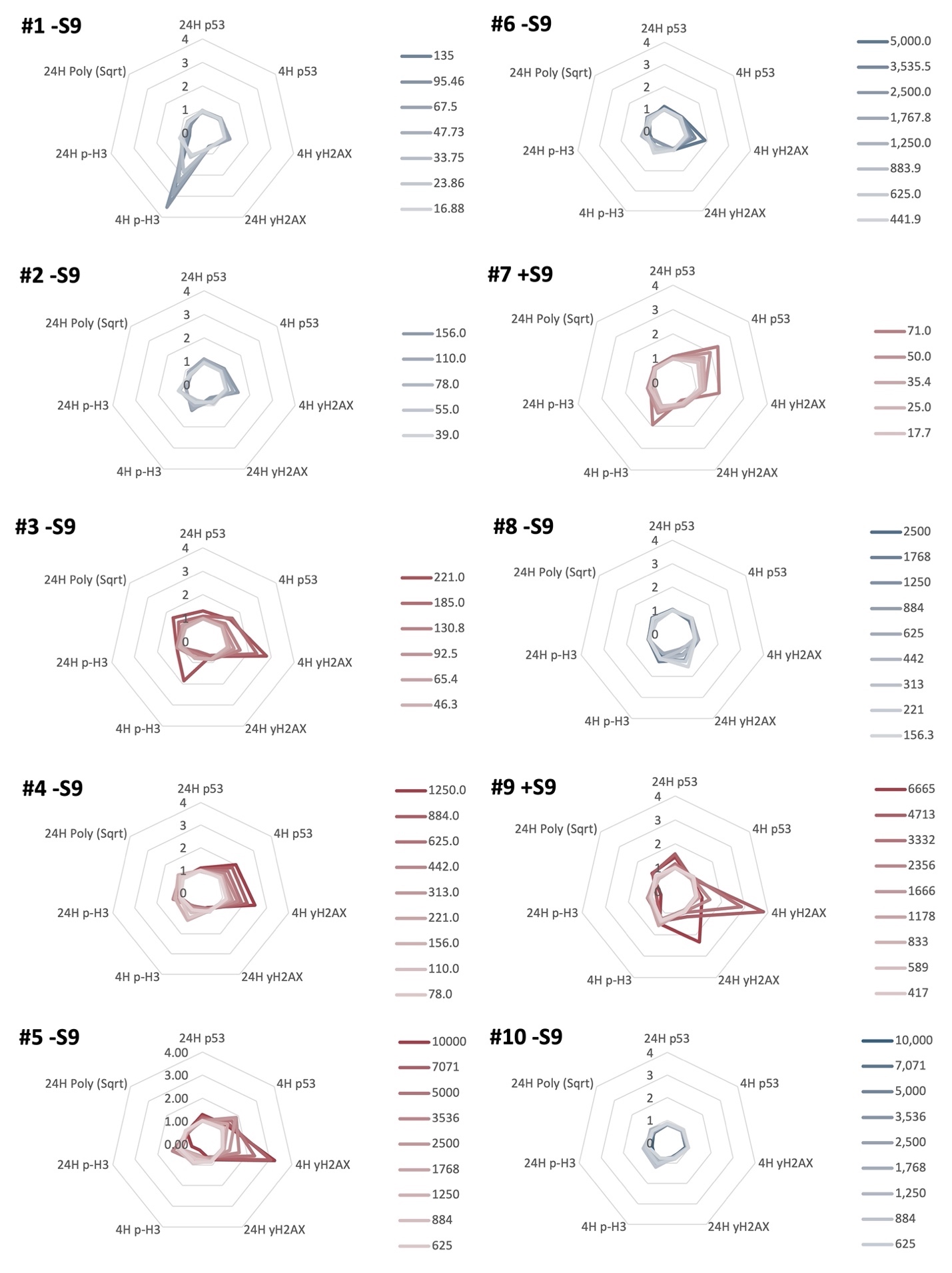


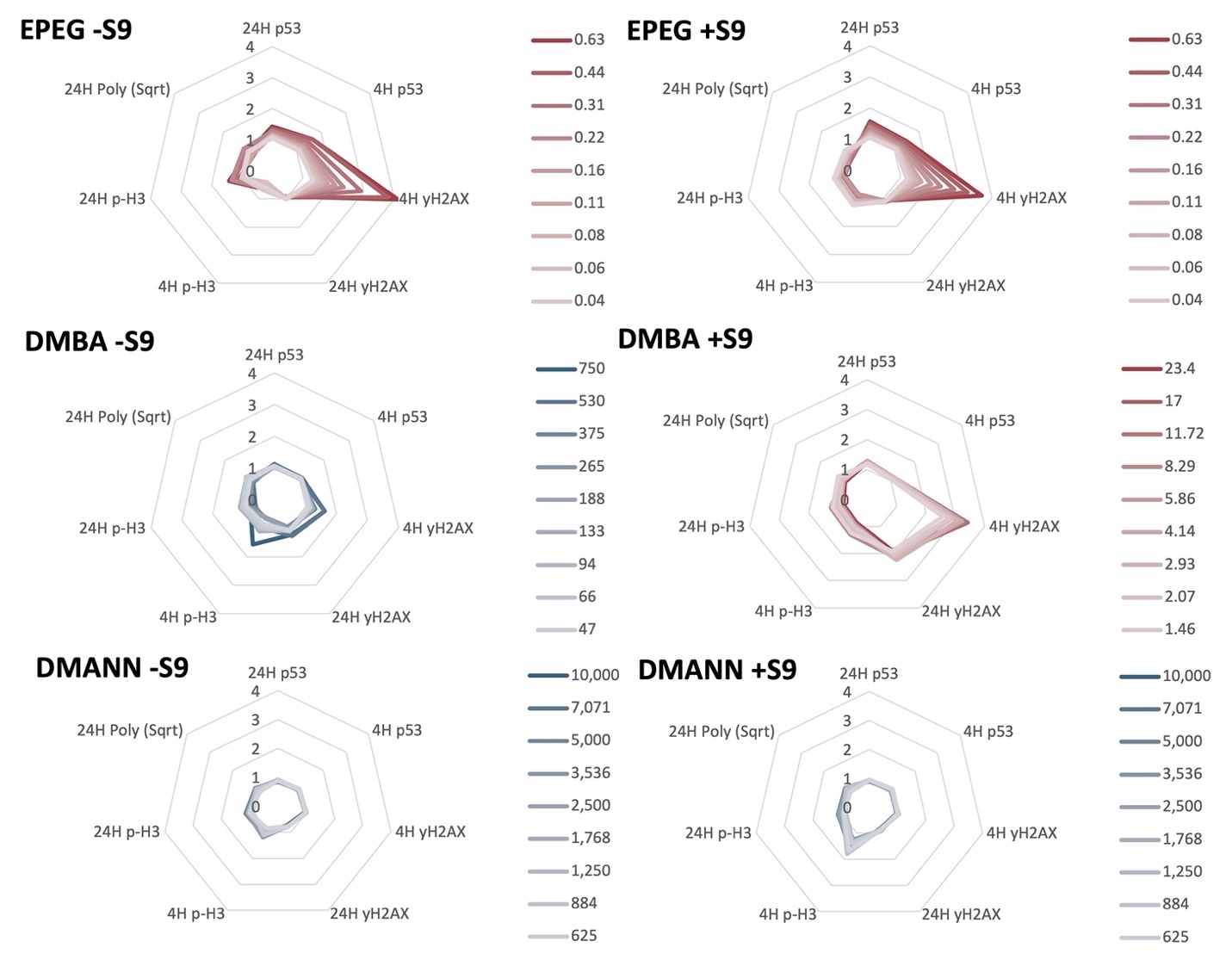


**Supplementary Figure 5: MultiFlow® DNA Damage assay classification results for ten NSACB data-poor substances (#1-10) and control chemicals.** Each radar plot shows the seven biomarkers predicting the predominant mode of action (MoA) for each chemical. Clastogen MoA biomarkers are on the right: 4H p53, 4H ɣH2AX, 24H p53, and 24H ɣH2AX. Aneugen MoA biomarkers are on the left: 4H p-H3, 24H p-H3, 24H Polyploidy, and 24H p53. The biomarker data are expressed as a fold-increase over the mean solvent control for each non-cytotoxic concentration (>20% viability) represented by lines with different colour intensities (as shown in the legend). The line colour in each plot represents the classification call: clastogens are red, non-genotoxicants are blue, and pan-genotoxicants are orange. Chemicals meeting or exceeding the Global Evaluation Factors (GEFs) in at least one concentration in two MoA-specific biomarkers were classified as aneugenic or clastogenic, or classified as pan-genotoxic if both the aneugen and clastogen criteria were met.


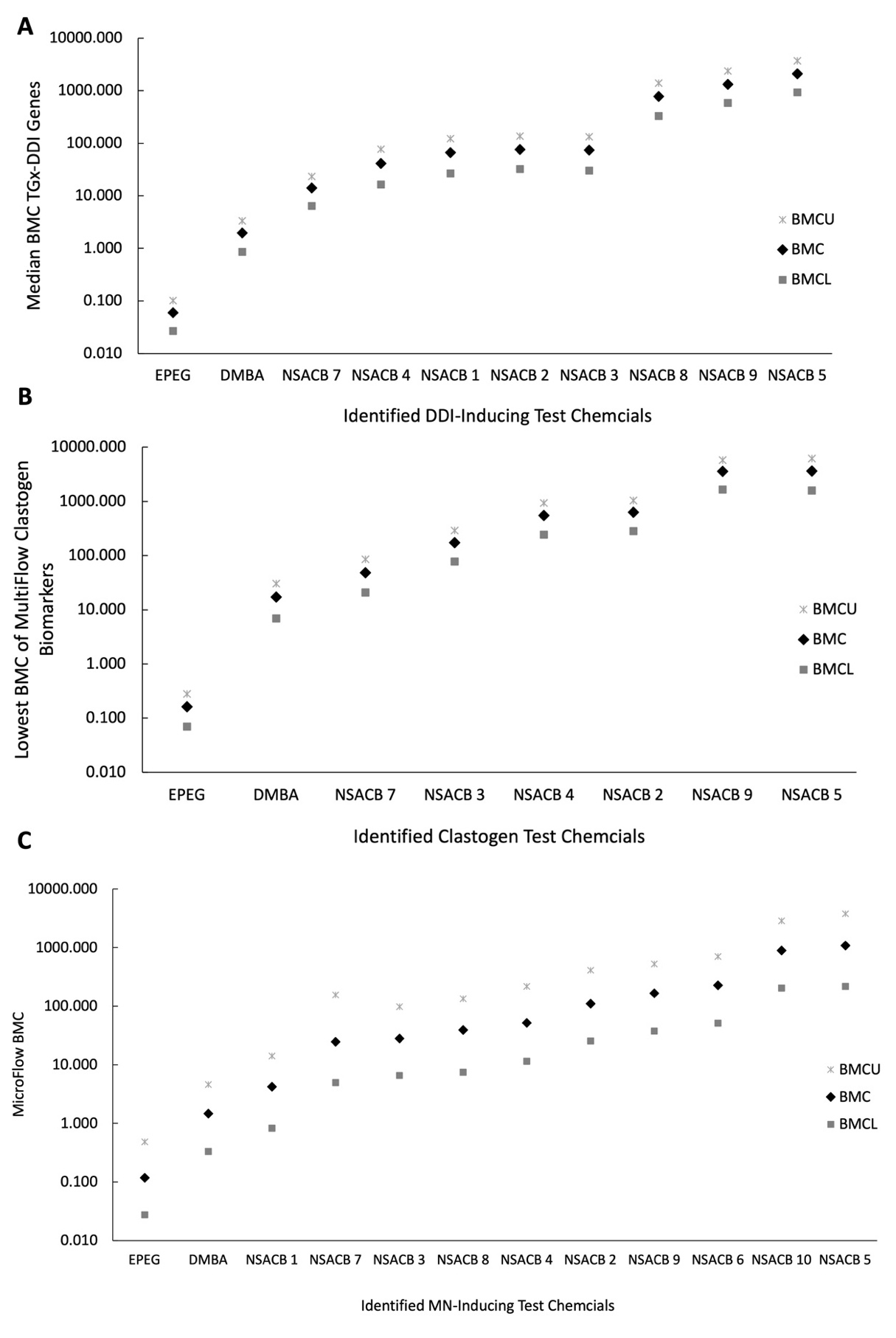


**Supplementary Figure 6: Comparison of potency ranking for the positive NSACB compounds from each assay based on their respective Benchmark Concentrations (BMCs).** (A) The potency ranking from the TGx-DDI transcriptomic biomarker based on median gene BMC, (B) the potency ranking from MultiFlow® assay based on the lowest clastogen biomarker BMC, and (C) the ranking from the in vitro MicroFlow® assay.


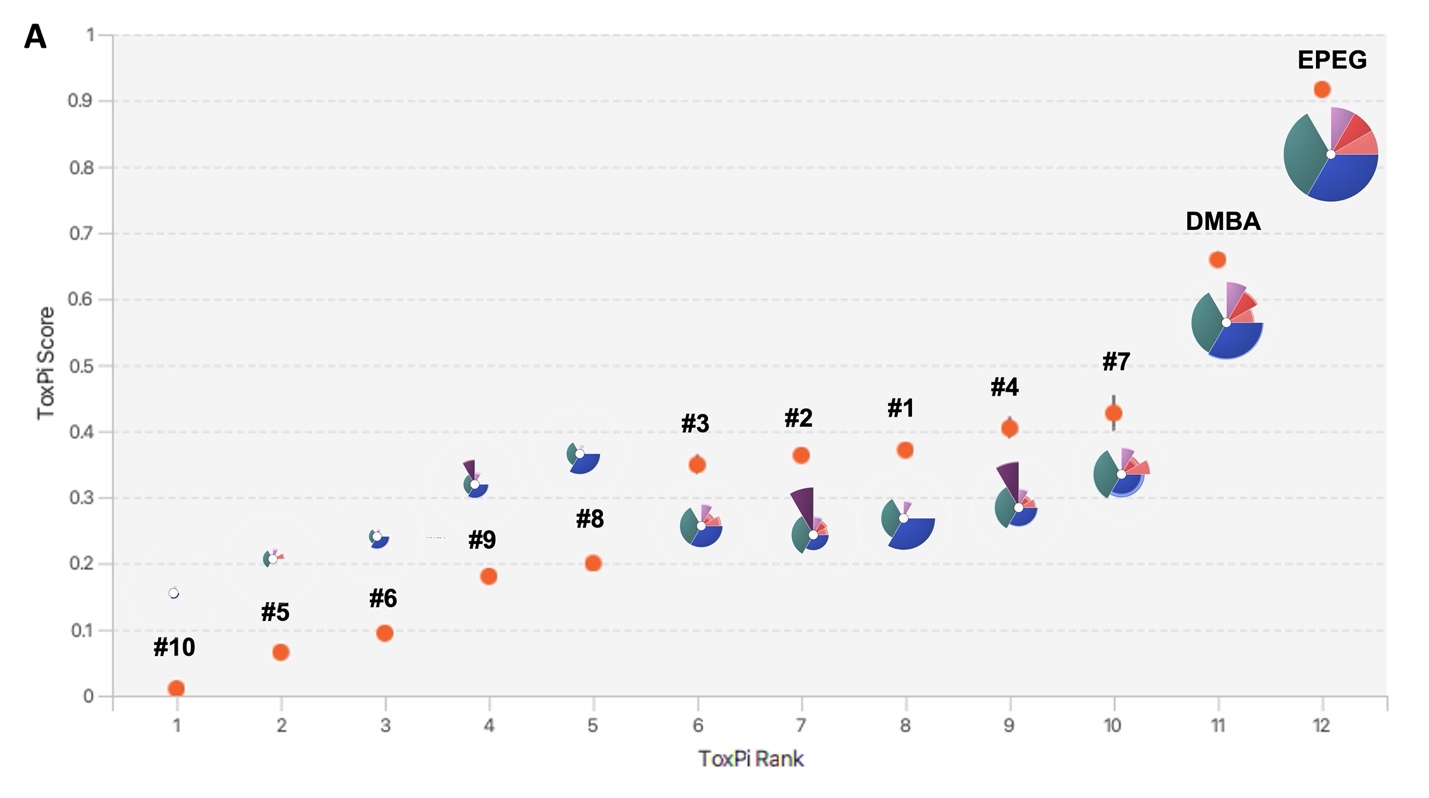


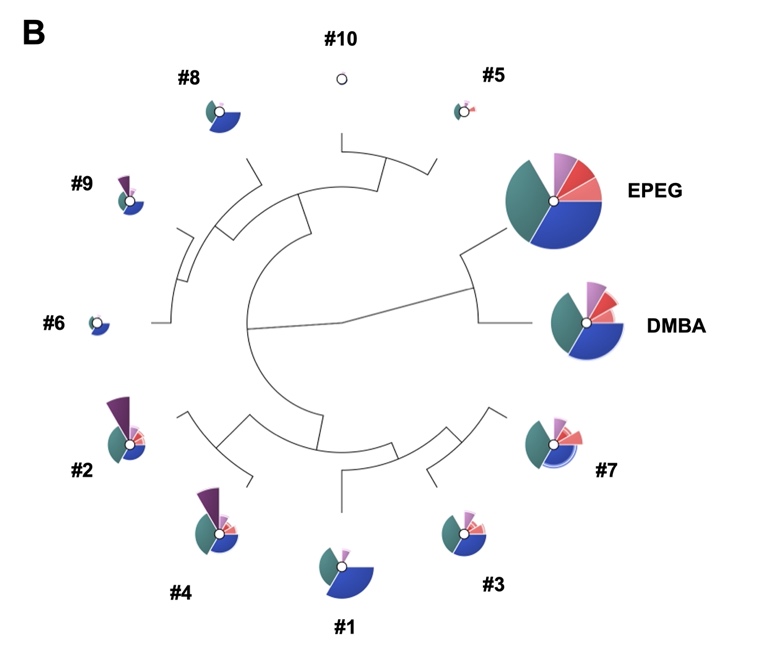


**Supplementary Figure 7: ToxPi visualization of multiplexed BMCs for the integrated test strategy.** (A) ToxPi score rankings and profiles for the data-poor compounds. For the ToxPi profiles, the distance of each slice from the origin indicates the slice score and endpoint potency (i.e., -log10 BMC). Slices represent the following endpoints: teal is TGx-DDI BMC, blue is MicroFlow® BMC, pink and purple are the MultiFlow® BMCs (i.e., dark pink is 24H p53, light pink is 4H p53, dark purple is 24H ɣH2AX, light purple is 4H ɣH2AX). Lower and upper bound confidence intervals are indicated by lighter shaded areas at the periphery of each slice. The width of each slice indicates the assigned endpoint weight. The TGx-DDI, MicroFlow®, and combined MultiFlow® endpoints each represent 1/3 of the profile. (B) Hierarchical clustering of the ToxPi profiles. The ToxPi algorithm groups substances with similar toxicological profiles.
